# Loss of ferroportin induces memory impairment by promoting ferroptosis in Alzheimer’s disease

**DOI:** 10.1038/s41418-020-00685-9

**Published:** 2021-01-04

**Authors:** Wen-Dai Bao, Pei Pang, Xiao-Ting Zhou, Fan Hu, Wan Xiong, Kai Chen, Jing Wang, Fudi Wang, Dong Xie, Ya-Zhuo Hu, Zhi-Tao Han, Hong-Hong Zhang, Wang-Xia Wang, Peter T. Nelson, Jian-Guo Chen, Youming Lu, Heng-Ye Man, Dan Liu, Ling-Qiang Zhu

**Affiliations:** 1 Department of Pathophysiology, Key Lab of Neurological Disorder of Education Ministry, School of Basic Medicine, Tongji Medical College, Huazhong University of Science and Technology, Wuhan, 430030 PR China; 2 The Institute of Brain Research, Collaborative Innovation Center for Brain Science, Huazhong University of Science and Technology, Wuhan, 430030 PR China; 3 Department of Radiology, Union Hospital, Tongji Medical College, Huazhong University of Science and Technology, Wuhan, Hubei PR China; 4 Department of Nutrition, School of Public Health, Zhejiang University, Hangzhou, Zhejiang 310058 PR China; 5 Institute of Nutritional Science, Shanghai Institutes for Biological Sciences, Chinese Academy of Sciences, 320 Yueyang Road, Shanghai, 200031 PR China; 6 Beijing Key Laboratory of Aging and Geriatrics, National Clinical Research Center for Geriatric Disease, Institute of Geriatrics, Chinese PLA General Hospital and Chinese PLA Medical Academy, Beijing, PR China; 7 Sanders Brown Center on Aging, Pathology and Laboratory Medicine, University of Kentucky, Lexington, KY 40536 USA; 8 Department of Biology, Boston University, Boston, MA 02215 USA

**Keywords:** Neural ageing, Ageing

## Abstract

Iron homeostasis disturbance has been implicated in Alzheimer’s disease (AD), and excess iron exacerbates oxidative damage and cognitive defects. Ferroptosis is a nonapoptotic form of cell death dependent upon intracellular iron. However, the involvement of ferroptosis in the pathogenesis of AD remains elusive. Here, we report that ferroportin1 (Fpn), the only identified mammalian nonheme iron exporter, was downregulated in the brains of APPswe/PS1dE9 mice as an Alzheimer’s mouse model and Alzheimer’s patients. Genetic deletion of Fpn in principal neurons of the neocortex and hippocampus by breeding Fpn^fl/fl^ mice with NEX-Cre mice led to AD-like hippocampal atrophy and memory deficits. Interestingly, the canonical morphological and molecular characteristics of ferroptosis were observed in both Fpn^fl/fl/NEXcre^ and AD mice. Gene set enrichment analysis (GSEA) of ferroptosis-related RNA-seq data showed that the differentially expressed genes were highly enriched in gene sets associated with AD. Furthermore, administration of specific inhibitors of ferroptosis effectively reduced the neuronal death and memory impairments induced by Aβ aggregation in vitro and in vivo. In addition, restoring Fpn ameliorated ferroptosis and memory impairment in APPswe/PS1dE9 mice. Our study demonstrates the critical role of Fpn and ferroptosis in the progression of AD, thus provides promising therapeutic approaches for this disease.

## Introduction

Iron is an essential element involved in many important biological processes in the central nervous system, such as oxygen transportation, myelin production, and the synthesis and metabolism of neurotransmitters [1]. High concentrations of iron are present in the brains of patients and transgenic mouse models of AD [2, 3]. Excess iron accumulates in the insoluble amyloid plaques and neurofibrillary tangles as characteristics of Alzheimer’s disease [4, 5]. Elevated neuronal iron exacerbates oxidative damage in neuronal cells and fosters multiple pathologies, ultimately producing pronounced cognitive deficits in AD [6–8]. Furthermore, there is an apparent link between an age-related elevation in iron load and the symptoms of AD [9]. Thus, abnormal iron homeostasis could contribute to the neuropathology of AD.

Ferroptosis is a nonapoptotic form of cell death dependent upon intracellular iron that is morphologically, biochemically, and genetically distinct from other forms of cell death, including apoptosis, necrosis, and autophagy [10]. Iron overload could induce ferroptosis in vivo, and ferroptosis could be specifically prevented using an iron chelator [10, 11]. Emerging evidence has suggested that ferroptosis could be discovered in the neuronal cell death associated with various neurological diseases, such as hemorrhagic stroke, ischemic stroke, Parkinson’s disease and Huntington’s disease, accompanied by lipid peroxidation, mitochondrial dysfunction and reduction of glutathione peroxidase 4 (GPX4) [12–16]. In addition, ferroptotic inhibitors have been shown to protect neurons and recover cognitive function in disease animal models of stroke [12, 15]. Knocking out GPX4 in mice directly resulted in age-dependent neurodegenerative changes and significant neuronal loss [17]. Considering the excess iron accumulation in AD, which induces significantly higher ROS production in the brain, ferroptosis is likely involved in the neuronal loss and cognitive impairment of AD. However, direct evidence is still lacking.

Ferroportin1 (Fpn, also known as solute carrier family 40 member 1, SLC40A1) is the only mammalian nonheme cellular iron exporter identified to date. It transports iron from iron storage cells into the blood to optimize systemic iron homeostasis [18]. Mice with global deletion of Fpn are embryonically lethal, suggesting the essential role of Fpn in development [19, 20]. In the central nervous system, Fpn is distributed in most cell types, including neurons, astrocytes, oligodendrocytes, and brain microvascular endothelial cells [21]. It is also essential for mouse embryonic development, forebrain patterning and neural tube closure [22]. Previous studies have shown that Fpn is likely downregulated in the brain tissues of AD patients and APPswe/PS1dE9 mice [23–25]. However, the precise role of Fpn in the brain iron deregulation and cognitive impairment of AD remains elusive.

Here, we reported that the expression of Fpn is decreased in the brains of an AD mouse model and AD patients, in which abnormal iron deposition has been observed. We generated conditional knockout mice (Fpn^fl/fl/NEXcre^) by crossing Fpn-floxed (Fpn^*fl/fl*^) mice with NEX-Cre mice that expressed Cre recombinase under the control of regulatory sequences of NEX, a gene that encodes a neuronal basic helix-loop-helix protein. NEX-cre mice are efficient tools for genetic targeting of principal neurons in the neocortex and hippocampus [26]. A series of studies were also conducted based on these Fpn-floxed mice [19], elaborating the function of Fpn1 in hepatocytes [27], macrophages [28], and red blood cells [18]. Conditional *Fpn ko* (Fpn^fl/fl/NEXcre^) mice in the excitatory neurons displayed AD-like hippocampal atrophy and memory loss. Moreover, we reported shrunken mitochondria and other ferroptosis phenotypes in an AD mouse model, and these changes were regulated by pathological Fpn loss in AD. Overexpression of Fpn in the hippocampus partially ameliorates the ferroptosis and memory impairments in the AD mouse model. Thus, we reasoned that elevation of Fpn or amelioration of ferroptosis might be a promising therapeutic approach for AD.

## Materials and methods

### Animals

Fpn-floxed (Fpn^fl/fl^) mice, which were described previously [19, 27, 28], were obtained from Dr. N.C. Andrews and transferred to the C57BL/6 (C57) background. NEX-Cre mice [26] were kindly provided by Dr Zilong Qiu of the Institute of Neuroscience, Chinese Academy of Sciences. APPswe/PS1dE9 mice were purchased from the Jackson Laboratory (Bar Harbor, ME, USA, Stock #034829) and bred in the Experimental Animal Central of Tongji Medical College, Huazhong University of Science and Technology. NEX-Cre mice were mated with Fpn-floxed (Fpn^fl/fl^) mice to generate conditional Fpn^fl/fl/NEXcre^ mice. All of the strains were maintained on a C57 background. Genotyping was performed as previously described [19, 26]. All of the mice were maintained in specific pathogen-free husbandry and fed a standard rodent diet. We allocate the mice into different group with their strains and the virus injected. About the different group with same strain, we allocate them randomly before different virus injected. The investigators were double-blinded to group allocation during data collection and analysis about the behavior tests and other experiments. All of the animal procedures followed guidelines and were approved by the Animal Care and Use Committee of Tongji Medical College under approval number 2016-S2189.

### Human brain samples

**Fig. 1:**
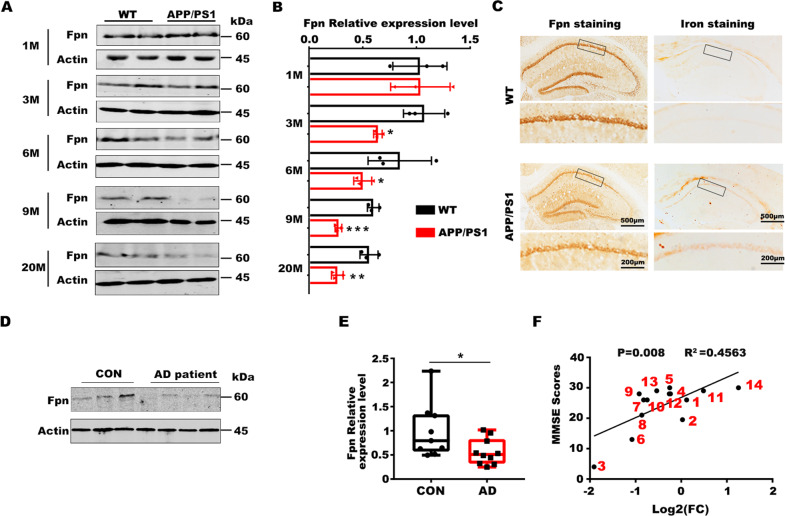
Fpn is downregulated in the hippocampus of AD mice and patient brain tissues. **A** Representive protein level of Fpn in the hippocampus of APPswe/PS1dE9 (APP/PS1) mice at different ages (M: month) and the age-matched wild-type littermates (WT). **B** The quantification for protein level of Fpn in the hippocampus of APPswe/PS1dE9 (APP/PS1) mice (*n* = 3). **C** Immuno-histochemistry of Fpn and the DAB-enhanced Perl’s Prussian blue iron staining in the hippocampus of APPswe/PS1dE9 (APP/PS1) mice and the age-matched wild-type littermates (WT) at 9 months old. **D** The protein level of Fpn in brain tissues of AD patients and control (CON) sample (frontal cortex, 3 con vs 4 AD). **E** The fold change of the quantification for the protein level of Fpn in cortical brain tissues of AD patients (*n* = 10) compared to corresponding control (CON) samples (*n* = 9). **F** Correlation analysis between the Fpn protein level (Log_2_FC: The log2 fold-change of the Fpn protein expression level compared to the average of the controls) and the MMSE scores of subjects. The corresponding serial numbers of the human samples are marked with red number adjacent to each point (*n* = 14). Protein expression levels were detected by western blotting. Data are shown as the mean ± SD of at least three independent experiments. Statistical analyses were carried out using two-way ANOVA and mutiple *t*-test. *p < 0.05; **p < 0.01; ***p < 0.001. Detailed Statistical analyses are included with the Supplementary Table S4.

Cortical tissues from the brains of nondementia control subjects and AD cases based on neuropathological diagnosis in Fig. 1D (3CON vs 4AD) was obtained from the Tissue Bank of the Institute of Geriatrics, Chinese PLA General Hospital and Chinese PLA Medical Academy. The patient information was described previously [29]. Cortical tissues (temporal pole) from the brains of nondementia control subjects and AD cases, based on neuropathological diagnosis in Supplementary Fig. S1D (7CON vs 7AD), were obtained from the University of Kentucky Alzheimer’s Disease Center (supported by NIH/NIA P30 AG028383) autopsy cohort. Detailed patient information is listed in Supplementary Table 1. Informed consents were obtained from all the subjects. The present study was approved by the ethics committee of Tongji Medical College (Wuhan, China).

### Western blot

Mice were decapitated and the brain tissues of hippocampus, cortex and other parts were immediately removed. The tissues were homogenated with sample buffer (pH 7.6, 50 mM Tris-HCl, 10 mM dithiothreitol, 2% sodium dodecyl sulfate, 10% glycerol, and 0.2% bromophenol blue) and protease inhibitors cocktail (Roche) on ice and boiled for 10 min. Proteins (10–50 µg) were separated by 10% SDS-PAGE gel and transferred to nitrocellulose membranes (Merck Millipore, Burlington, MA, USA). The membranes were incubated with primary antibodies overnight at 4 °C followed by incubation with anti-rabbit or anti-mouse IgG conjugated secondary antibodies (LI-COR, Lincoln, NE, USA) for 1 h at room temperature, and detected using the Odyssey Imaging System (LI-COR, Lincoln, NE, USA). Primary antibodies used were: Slc40A1 (Fpn1, Novus Biologicals, CO, USA); Slc40A1 (Fpn1, Alphadiagnosis, San Antonio, TX, USA); FTH (FTH1, ferritin heavy chain, Cell Signaling Technology, Danvers, MA, USA), Gpx4; actin (Proteintech, Wuhan, China). Detailed information about the antibodies is available in Supplementary Table S2.

### Quantitative RT-PCR

Total RNA was extracted by TRIzol reagent (Invitrogen, CA, USA). One microgram of RNA was reverse transcribed into cDNA using a first strand cDNA synthesis kit (Toyobo, Osaka, Japan) according to the manufacturer’s instructions. RNA extraction and reverse-transcription were performed as described previously [30]. qRT-PCR was performed on an ABI StepOne Plus using SYBR Green^®^ Premix Ex Taq (Takara, Tokyo, Japan). Expression levels were normalized against β-actin. The set of deltaCq replicates (Cq values for each sample normalized against the geometric means of the reference genes) for control and tested samples were used for statistical testing and estimation of the *p* values. Shown are fold changes versus untreated controls. The primers were purchased from Tsingke Biological Technology (Beijing, China), and the sequences are detailed in Supplementary Table S3.

### Immunohistochemistry

Mice were anesthetized using a mixture of ketamine (100 mg/kg) and dexmedetomidine (0.5 mg/kg) and immediately perfused with normal saline and 4% paraformaldehyde solution continuously. The brains were dissected and postfixed for 24 h at 4 °C, and 25-μm slices of brain tissues were cut with a vibrate. For immunohistochemistry, the slices were incubated with Fpn primary antibody (Alphadiagnosis, San Antonio, TX, USA catalog MTP11-A, 1:100) for 48 h, probed with biotin-labeled secondary antibodies (1:10,000) for 1 h at 37 °C, and detected with the DAB Kit (Zsbio, Beijing, China, Catalog ZLI-9018). The images were observed under a microscope (Olympus BX60, Tokyo, Japan). DAB-enhanced Perl’s staining was used to detect iron accumulation as previously described [19]. Sections of brain tissue were washed with PBS and incubated in freshly prepared Perls solution (5% potassium ferrocyanide [Sigma-Aldrich, St. Louis, MO, USA]/10% hydrochloric acid) for 1 h, endogenous peroxidase activity was quenched for 20 min at room temperature in 0.3% H_2_O_2_ in methanol, followed by washing with PBS five times. Then, DAB incubation was carried out for 3 min. Nissl staining was performed using the Nissl Staining Kit (Beyotime Technology, Shanghai, China, catalog C0117) according to the instructions of the manufacturer. The neuron-counting procedure was performed by ImageJ software, as described in our previous study [31]. Three relative coronal plane sections from each brain across the hippocampus were analyzed, and the average number of the three sections was regarded as the data for each sample. For each experimental group, brains from five mice were stained and analyzed.

### Stereotaxic injection and drug administration

For stereotaxic injection, mice were anesthetized using a mixture of ketamine (100 mg/kg) and dexmedetomidine (0.5 mg/kg). Holes were drilled above the DG field of the hippocampus (bregma: anterior/posterior −2.0 mm, medial/lateral ±1.2 mm, and dorsal/ventral −2.2 mm). Adeno-associated viruses (AAVs) (10^12^ IU/ml, 2 μl) for Fpn overexpression or lentiviruses (10^9^ IU/ml, 2 μl) for Fpn knockdown and the relative control viruses were bilaterally microinfused into the hippocampus via a cannula connected to a Hamilton microsyringe (Reno, NV, USA). We injected the AAV packaged full length murine Fpn cDNA into the hippocampus of APPswe/PS1dE9 mice at 9 months old. 3 months later, behavioral tests were performed in these mice at 12 months old. The infusion rate was 0.2 μl/min, and the cannula was left in place for 10 min following completion of the infusion. AAVs for Fpn overexpression were purchased from Neuron Biotech (Shanghai, China), and lentivirus for Fpn short hairpin RNA was purchased from Genechem Co, Ltd. (Shanghai, China). Oligomeric Aβ_1–42_ was injected into the mouse hippocampus as previously reported [32]. Briefly, 100 µM oligomeric Aβ_1–42_ (Chinese Peptide Company, Hangzhou, China, Catalog AMYD- 002) was prepared and incubated for 12 h at 4 °C before injection, and 4 µl of Aβ_1–42_ were bilaterally injected into the dentate gyrus at a rate of 0.5 µl/min. The procedure for stereotaxic injection was consistent with the virus injection. Aβ_1–42_ was injected only once. Liproxstatin (5 mg/kg, Selleck Chemicals, Houston, TX, USA, Catalog S7699), ferrostatin-1 (5 mg/kg, Sigma, St. Louis, MO, USA) or vehicle (18.8% DMSO in 0.9% saline) were delivered intranasally by pipette to the mice once per day since the day of Aβ_1–42_ exposure till the mice were sacrificed. The Morris water maze was performed on these mice after treatment of drugs for 1 week. On the days of Morris water maze testing, drug administration was performed at 1 h after the test. Finally, the mice were sacrificed, and the brain tissues were immediately removed for the further experiments (Western blot, immunohistochemistry), as previously described [15].

### Morris water maze

The Morris water maze was performed as previously described [33]. Briefly, mice were trained for five consecutive days to find a platform hidden 1 cm under water using a stationary array of cues on the walls. A digital tracking device was connected to a computer and was used to track the movement of the mice in the pool. The escape latency of the mice in reaching the hidden platform was detected every day. On the seventh day, the hidden platform was removed. The latency in reaching the place of the former platform (the time spent before the first crossing of the place of platform) and swimming velocity were measured, and the percentage of time spent in the target quadrant was also recorded.

### Contextual and cue fear conditioning

The experimental device and recording software [FCT-100] were purchased from Tai Meng Technology Co., Ltd. (Chengdu, China). Before training, the mice were placed into a single chamber (33 × 33 × 35 cm) with a metal grid at the bottom for 5 min to adapt to the novel environment, and the number and percentage of freezing behaviors were recorded. On the training day, the mice were subjected to 5-min trials that began with a 30-s tone, followed by a 2-s foot shock (1 mA) and then a 30-s interval, which was repeated three times (freezing time during training). After 24 h, contextual and tone conditioning tasks were assessed. To test contextual conditioning fear, the mice were returned to the same chamber, and contextual learning was assessed during the same procedure as on training day except for the foot shock and tone (freezing time/context). To test tone-dependent conditioning fear, the environment in the chamber was changed (visual, tactile, and olfactory cues) to present the mice with a new context for testing. Then, the mice were placed in the chamber for 5 min (freezing time before tone), and the same procedure was followed as on training day except for the foot shock (freezing time/cue).

### Open field test

The open field test chamber was a rectangular chamber (60 × 60 × 40 cm) composed of gray polyvinyl chloride. The center area was illuminated by 25-W halogen bulbs (200 cm above the field). The mice were gently placed in one corner of the testing chamber and were allowed 5 min of free movement, which was monitored by an automated video tracking system. Images of the activities during these 5 min were automatically analyzed using the DigBehv animal behavior analysis program; the static time (Static T), moved time (Move T), moved distance (Move D) and the time spent at the center (Center T), corner (Corner T), and side (Side T) by the mice in this procedure were recorded and analyzed.

### Magnetic resonance imaging (MRI)

All the MRI examinations were performed with a 3.0-T MRI system (Discovery W750, GE) with a small animal coil. The protocol included axial fast-spin echo (FSE) T2-weighted imaging and an axial T1-weighted examination. A transverse T2-weighted image was obtained using the FSE sequence (repetition time/echo time [TR/TE], 3000/100 ms; field of view [FOV], 60 × 60 mm, section thickness, 2 mm; matrix size, 240 × 182; NSA, 5; intersection gap, 0.2 mm; and bandwidth = 257.8 Hz/pixel). The following parameters were used for this sequence: TR/TE = 2700/100 ms; FOV = 60 × 60 mm; section thickness = 2 mm; matrix size = 88 × 75; NSA = 5; intersection gap = 0.2 mm; bandwidth = 17.8 Hz/pixel; parallel imaging SENSE factor = 2.5 and number of slices = 10.

### Measurement of iron indices and serum parameters

Quantitative measurement of tissue nonheme iron was performed using the method of Torrance and Bothwelld [27, 34]. The results are presented as micrograms of iron per gram wet weight of tissue. Serum iron concentrations were determined using a serum iron assay kit (Nanjing Jiancheng Bioengineering institute, Nanjing, China, Catalog A040-1-1). Total iron binding capacity (TIBC) was determined using a total iron binding capacity assay kit (Nanjing Jiancheng Bioengineering institute, Nanjing, China, Catalog A039-1-1), according to the manufacturer’s instructions. Transferrin saturation was directly calculated from the serum iron and TIBC.

### Measurement of GSH and MDA levels

Mice were anesthetized using a mixture of ketamine (100 mg/kg) and dexmedetomidine (0.5 mg/kg) and then decapitated, and the hippocampus was immediately removed. The fresh tissues of hippocampus were perfused with PBS containing heparin to remove blood and clots. After weighing the tissue, it was homogenized in PBS containing 2 mM EDTA. The glutathione (GSH) level was determined using a GSH-Glo™ Glutathione Assay kit (Promega, Madison, WI, USA, Catalog V6911). The MDA content was determined using an MDA detection kit (Beyotime Technology, Shanghai, China, catalog S0131), according to the manufacturer’s instructions.

### Transmission electron microscopy (TEM)

Mice were anesthetized using a mixture of ketamine (100 mg/kg) and dexmedetomidine (0.5 mg/kg) and immediately perfused with normal saline and 4% paraformaldehyde solution continuously. Then, the brains of Fpn^fl/fl^, Fpn^fl/fl/NEXcre^, WT, and APPswe/PS1dE9 mice at 9 months of age were fixed in 2% glutaraldehyde at 4 °C overnight. Subsequently, the hippocampus was dissected. Tissues were washed with cacodylate buffer, postfixed in 1% OsO_4_ for 2 h, washed with cacodylate buffer, placed in 1% uranylacetate for 1 h, and dehydrated in ethanol. The tissues were then infiltrated through a propylene oxide/Epon series, embedded in Epon, sectioned with an ultramicrotome (Leica, Weztlar, Germany), and placed on EM grids. Sections were stained with uranyl acetate and lead citrate and then examined with a Hitachi HT-7700 electron microscope.

### Primary neuron culture and treatment

Mice primary hippocampal neurons were isolated as previously reported [35]. Briefly, the hippocampi of the embryonic mice (C57) at E19 were collected and incubated with 0.25% trypsin in D-Hanks for 15 min. Then, neuronal plating medium containing DMEM/F12 with 10% FBS was added to the hippocampi, and then they were centrifuged at 1000 × *g* for 5 min. The cells were triturated and plated onto a plastic culture dish and incubated at 37 °C with 5% CO_2_. After 7 days in vitro, recombinant human Aβ_1–42_ (Chinese Peptide Company, Hangzhou, China, Catalog AMYD-002) was resuspended in DMSO to 5 mM as described previously [36], and 5 mM Aβ_1–42_ was diluted with sterile (1×) PBS to 100 µM on the day before treatment. Then, Aβ_1–42_ solution was incubated for 24 h at 4 ˚C. After incubation, the Aβ_1–42_ solution was diluted into concentrations indicated for treatment with DMEM/F12. The neurons were treated with 10 μm/20 μm Aβ_1–42_ as a lethal concentration for 24 h, and vehicle treatment was used for controls. Treatment with 100 nM Liproxstatin (Selleck Chemicals, Houston, TX, USA, Catalog S7699) or 1 µM ferrostatin-1 (Sigma-Aldrich, St. Louis, MO, USA, Catalog SML0583) with Aβ_1–42_ was used to test the effects of ferroptosis inhibitors on the cytotoxicity of Aβ_1–42_. Inhibitors of apoptosis (10 μM, Emricasan, Selleck Chemicals, Houston, TX, USA, Catalog S7775) or necrosis (50 μM, Nec-1, Merck Millipore, Darmstadt, Germany, Catalog 504297) were also used as controls.

### Assessment of neuronal viability in neuronal cultures

Analysis of neuronal survival was measured with a CCK8 assay (YEASEN, Wuhan, China, Catalog 40710ES03) and PI staining (YEASEN, Wuhan, China, Catalog 40755ES64), according to the instructions of the manufacturer. Primary neuron cells were washed with the medium after treatment, and then CCK8 was added to the medium of the neuronal cultures. After 2 h at 37 °C, the plates were directly spectrophotometrically quantified at 450 nm. Data are presented as percentages of neuronal viability relative to the control conditions at a 100% value. For PI staining, cells were washed with the medium after treatment and then incubated with 5 μg/ml PI for 20 minutes. The cells were washed with PBS and fixed in 4% paraformaldehyde for 20 min and then stained with DAPI for 15 min. The cell number was measured with ImageJ software (NIH), and the percentage of cell death is expressed as PI + cells/DAPI + cells per ×200 field; ten fields from 3–5 mice/per groups were calculated.

### Statistics and data collection

Data are shown as the mean ± SD/SEM of at least three independent experiments. Statistical significance was considered at *p < 0.05, **p < 0.01, ***p < 0.001. Unpaired t-tests (two-tailed) were used for single comparisons, and two-way ANOVA was used for multiple comparisons. For each experiment, detailed statistical analysis and sample size (*n* number) are carefully reported in figure legends and in Supplementary Table S4. Statistical analyses were performed in GraphPad Prism software, version 6.0, and Microsoft Excel software. The investigator was blinded to the group allocation during the experiment and data collection.

## Results

### Fpn is downregulated in the hippocampus of *APPswe/PS1dE9* mice and brain tissues of AD patients

To explore the role of Fpn in the pathogenesis of AD, we first examined the expression levels of Fpn in the brains of AD mice and patients. We observed an age-dependent downregulation of Fpn at the protein level in the hippocampus and frontal cortex of APPswe/PS1dE9 (APP/PS1) mice compared with wild-type littermates (Fig. 1A, B and Supplementary Fig. S1A). The loss of Fpn was accompanied by brain iron overload in the hippocampus of AD mice at 9 months old (Fig. 1C). In the brain tissues of AD patients, Fpn protein was also significantly decreased (Fig. 1D, E and Supplementary Fig. S1D). The analysis of Fpn protein levels and the MMSE (Mini-mental State Examination) scores of the patients revealed that lower Fpn protein levels were associated with more serious MMSE scores (Fig. 1F), indicating involvement of Fpn in cognitive impairment in AD. The mRNA level was not affected in the AD mice (Supplementary Fig. S1B, C), and in AD patient samples (Supplementary Fig. S1E), suggesting that Fpn undergoes posttranscriptional regulation in AD. These data indicate that Fpn is downregulated in AD. Besides, the content of tissue iron and FTH (Ferritin heavy chain) were also elevated in the hippocampus of APP/PS1 mice with age, which were consistent with the pathological downregulation of Fpn level in these tissues (Supplementary Fig. S2).

### Deficiency of *Fpn* in excitatory neurons induced brain atrophy and cognitive impairment

**Fig. 2:**
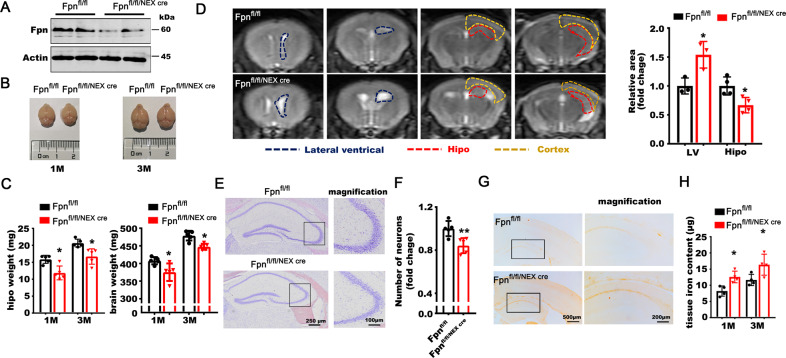
*Fpn*^*fl/fl/NEXcre*^ mice developed brain atrophy. **A** The protein level of the Fpn in the primary neurons hippocampus of Fpn^fl/fl/NEXcre^ mice. **B** The representative images of the whole brain of Fpn^fl/fl/NEXcre^ mice and age-matched floxed littermates (Fpn^fl/fl^). **C** The weight of the hippocampus and whole brain in Fpn^fl/fl/NEXcre^ mice (*n* = 5) and age-matched controls (Fpn^fl/fl^) (*n* = 5) littermates at 1 month and 3 month of age. **D** The representative MRI images (left) and relative quantitative data (right) from the brains of Fpn^fl/fl/NEXcre^ mice and age-matched wild type littermates at 1 month old (*n* = 3–4). **E** Nissl staining of Fpn^fl/fl/NEXcre^ mice at 3 month of age (Right panels are the magnification image as indicated in the left panels). **F** Quantitative fold change of the number of neurons in hippocampus by Nissl staining of Fpn^fl/fl/NEXcre^ mice (*n* = 5) and age-matched wild type littermates (*n* = 5) at 3 months old. **G** DAB-enhanced Perl’s Prussian blue iron staining of Fpn^fl/fl/NEXcre^ mice brains at 3 months old (right panels are the magnified images as indicated in the left panels). **H** The tissue iron content (μg/g) in the hippocampus of Fpn^fl/fl/NEXcre^ mice at 1 month old and 3 months old (*n* = 4). Protein expression levels were detected by western blotting. Data are shown as the mean ± SD of at least three independent experiments. Statistical analyses were carried out using two-way ANOVA and multiple *t*-tests. *p < 0.05; **p < 0.01; ***p < 0.001. Detailed Statistical analyses are included with the Supplementary Table S4.

To determine the precise role of Fpn in brain iron metabolism and cognitive impairment, we generated conditional knockout mice of Fpn (Fpn^fl/fl/NEXcre^) by crossing the Fpn^fl/fl^ mice with NEX-Cre mice, which expressed Cre recombinase in the excitatory neurons of the neocortex and hippocampus. The floxed littermates (Fpn^fl/fl^) of the KO mice were used as control animals. Fpn levels were significantly decreased in the hippocampus and neocortex of Fpn^fl/fl/NEXcre^ mice (Fig. 2A and Supplementary Fig. S3C) but not in other organs (Supplementary Fig. S3B, C). The Fpn^fl/fl/NEXcre^ mice displayed comparable body weights to their littermate floxed controls (Supplementary Fig. S4); however, the weight of the whole brain was reduced, particularly the hippocampus (Fig. 2B, C). The weight of the hippocampus was decreased by 10–30% at 3 months old (Fig. 2C). The MRI scans further revealed atrophy of the hippocampus, accompanied by enlargement of the lateral ventricle and noticeable thinning of the cortex (Fig. 2D and Supplementary Fig. S5). In addition, brain atrophy initially appeared in the hippocampus at 1 month of age (Fig. 2D) and became more apparent at 3 months of age (Supplementary Fig. S5). Nissl staining showed that visible neuronal loss occurred in the hippocampus of Fpn^fl/fl/NEXcre^ mice (Fig. 2E, F). Our results indicate that deficiency of Fpn in the neocortex and hippocampus could induce brain atrophy, especially in the hippocampus. It is known that progressive brain atrophy is one of the most prominent pathological hallmarks in AD [37]. Thus, downregulation of Fpn in AD was likely involved in brain atrophy and the pathogenesis of the disease.

To elucidate the disturbance of iron metabolism in Fpn^fl/fl/NEXcre^ mice, we measured the serum iron parameters and tissue nonheme iron contents. We found that the tissue nonheme iron levels in the hippocampus and cortex of Fpn^fl/fl/NEXcre^ mice were increased from 1 month old and 3 months old (Figs. 2G, H and S6A) separately. No difference was found in other parts of the brain (e.g., cerebellum) or other peripheral organs (Supplementary Fig. S6B, C). In line with this finding, the iron levels in the serum were also comparable to those in their littermate controls (Supplementary Table S5).

**Fig. 3:**
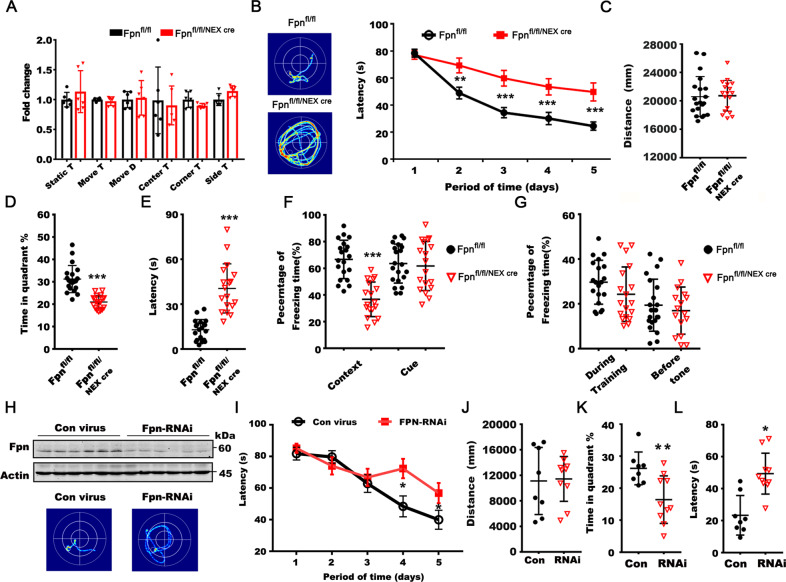
*Fpn*^*fl/fl/NEXcre*^ mice developed cognitive impairment. **A** The static time (Static T), moved time (Move T), moved distance (Move D) and the time spent at the center (Center T), corner (Corner T), side (Side T) of Fpn^fl/fl/NEXcre^ mice (*n* = 6) and age-matched wild type (WT) littermates (*n* = 6) in an open field test. **B** The representative searching trace (left) and the latency in the learning stages in the Morris water maze of Fpn^fl/fl/NEXcre^ mice (*n* = 18) and age-matched floxed littermates at 10–12 months old (*n* = 20). **C** The moved distance of *Fpn ko* mice (*n* = 18) and littermate control (*n* = 20) in Morris water maze. **D** The time in the target quadrant and (E) as well as the latency to reach a hidden platform on day 7 of the ko mice. **F** Decreased time spent freezing in the contextual fear conditioning in Fpn^fl/fl/NEXcre^ mice (Fpn^fl/fl^, *n* = 20, Fpn^fl/fl/NEXcre^, *n* = 18). **G** The percentage time spent freezing during training or before tone in the fear conditioning test of these mice. **H** The Fpn protein level in hippocampus of C57 mice injected with lentivirus expressing shRNA against fpn (FPN-RNAi) or scrambled hairpin (Con virus). **I** The representative searching trace (left) and the latency in the learning stages (right) in the Morris Water Maze test of C57 mice injected with lentivirus expressing shRNA against fpn (FPN-RNAi) (*n* = 10) or scrambled hairpin (Con virus) (*n* = 8). **J** The moved distance of these mice in Morris water maze tests. **K** The time spent in the target quadrant and (L) the latency to reach a hidden platform on day 7 of these mice. Data are shown as the mean ± SD of at least three independent experiments. Statistical analyses were carried out using two-way ANOVA and mutiple *t*-tests. *p < 0.05; **p < 0.01; ***p < 0.001. Detailed Statistical analyses are included with the Supplementary Table 4.

We next performed functional tests on learning and memory in Fpn^fl/fl/NEXcre^ mice. No significant differences were found in basal locomotive behavior between the KO mice and littermate controls (Fig. 3A, C). In Morris water maze tests, we found that the KO mice showed significantly worse learning performance during the training sessions (Fig. 3B). In the probe trial, the KO mice displayed reduced accuracy, prolonged latency in finding the target platform, and a shorter duration in the target quadrant (Fig. 3D, E). Additionally, Fpn^fl/fl/NEXcre^ mice spent remarkably less freezing time in a contextual fear memory task but not in cue fear memory, indicating impaired hippocampus-related learning/memory (Fig. 3F). No significant differences were observed in sensitivity to electrical shocks between the two groups (Fig. 3G). To avoid the possible developmental deficits caused by the genetic deletion of Fpn, we injected lentivirus packaged with a shRNA directed against Fpn into the hippocampus of 3-month-old C57 mice to knockdown the expression of Fpn in wild-type mice (Figs. 3H and Supplementary Fig. S7). The behavior test was performed 2 months after the virus injection. Similar to the Fpn^fl/fl/NEXcre^ mice, these mice displayed impaired spatial learning and memory retention on Morris water maze tests (Fig. 3H–L). These findings demonstrated that Fpn deficiency induce impairments in hippocampus-dependent memory, as seen in AD.

### The neurons in the hippocampus *of Fpn*^*fl/fl/NEXcre*^ and AD mice developed morphological and molecular features of ferroptosis

**Fig. 4:**
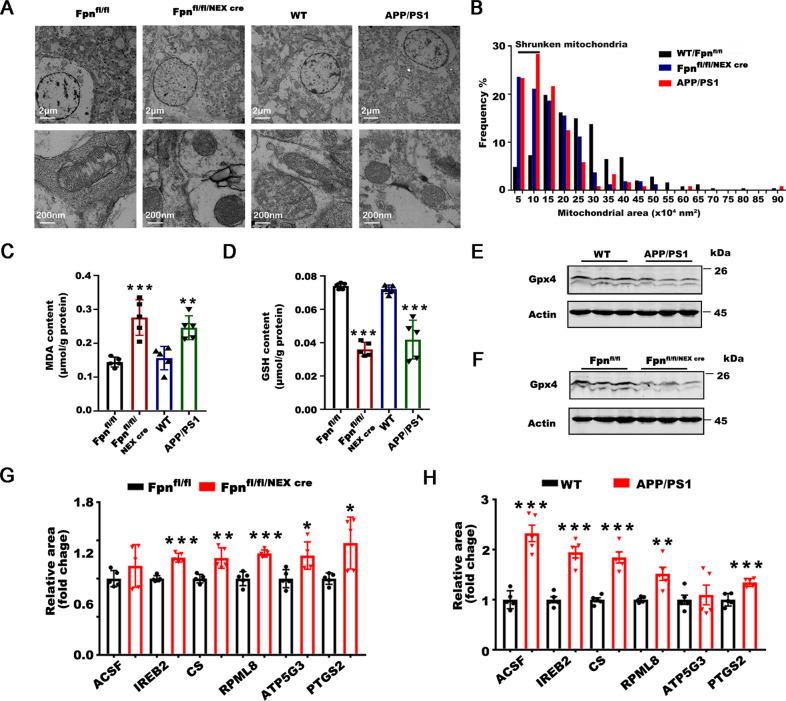
Both *Fpn*^*fl/fl/NEXcre*^ and AD mice developed features of ferroptosis. **A** Transmission electron microscopy pictures of perinuclear area of hippocampal neurons from Fpn^fl/fl^, Fpn^fl/fl/NEXcre^, WT and APPswe/PS1dE9 mice at 9 months old. WT were age-matched wild type littermates of the transgenic mice. **B** Mitochondrial area frequency in perinuclear compartment of these mice. Calculated from *n* > 100 mitochondria from *n* > 10 pictures of 3 mice per group. **C** The MDA content and **D** the GSH content in the hippocampus of Fpn^fl/fl^, Fpn^fl/fl/NEXcre^, WT and APPswe/PS1dE9 mice at 9 months old. WT were age-matched wild type littermates of the transgenic mice (*n* = 5). **E** The protein levels of Gpx-4 were detected in the hippocampus of the WT and APPswe/PS1dE9 mice and **F** Fpn^fl/fl^, Fpn^fl/fl/NEXcre^ mice. β-Actin was served as a loading control. **G** The mRNA level of ACSF, IREB2, CS, RPL8, ATP5G3 and PTGS2 were detected in the hippocampus of the WT (*n* = 5) and APPswe/PS1dE9 mice (*n* = 5) and **H** Fpn^fl/fl^ (*n* = 5), Fpn^fl/fl/NEXcre^ mice (*n* = 5). β-Actin was used as an internal control, and results are shown as fold change of the control. Data are shown as the mean ± SD of at least three independent experiments. Statistical analyses were carried out using two-way ANOVA and mutiple *t*-tests. *p < 0.05; **p < 0.01; ***p < 0.001. Detailed Statistical analyses are included with the Supplementary Table S4.

The neuronal loss (Fig. 2C–F) in the brains of Fpn^fl/fl/NEXcre^ mice raised the question of whether ferroptosis, a newly described iron-dependent cell death mechanism, is involved in the neuronal death in AD. First, we employed transmission electron microscopy to examine the ultrastructure of neurons from the hippocampus of Fpn^fl/fl/NEXcre^ and APPswe/PS1dE9 mice at 9 months old. We found smaller, ruptured mitochondria (the most prominent characteristic of ferroptosis) [38] in the perinuclear and cytoplasmic compartments of the hippocampal neurons in both Fpn^fl/fl/NEXcre^ and APPswe/PS1dE9 mice (Fig. 4A and Supplementary Fig. S8). The quantitative data showed that the frequency of smaller mitochondrial area was obviously increased in both murine models (Figs. 4B and S8B). Consistent with the shrunken mitochondria observed in these two models, both Fpn^fl/fl/NEXcre^ and APPswe/PS1dE9 mice had higher MDA levels, as well as lower GSH content in hippocampal tissue than did the Fpn-floxed and wild-type controls (Fig. 4C, D). To further verify ferroptosis involvement of neuronal death in these two murine models, we examined the expression of specific ferroptosis-related genes in these mice, including GPx4 [38], acyl-CoA synthetase family member 2 (ACSF2), citrate synthase (CS), iron response element binding protein 2 (IREB2), ribosomal protein L8 (RPL8), ATP synthase F0 complex subunit C3 (ATP5G3) [10], and prostaglandin-endoperoxide synthase 2 (PTGS2) [38]. Among those genes, GPx4 (a ferroptosis regulator) was downregulated, in both Fpn^fl/fl/NEXcre^ and APPswe/PS1dE9 mice (Fig. 4E, F) compared with littermate controls. Furthermore, the mRNA levels of IREB2, CS, RPL8, and PTGS2 were upregulated in both murine models, but ACSF2 transcript was increased only in APPswe/PS1dE9 mice and ATP5G3 was increased only in Fpn^fl/fl/NEXcre^ mice (Fig. 4G, H). All of these data suggested that ferroptosis is activated in the hippocampus of both Fpn^fl/fl/NEXcre^ and APPswe/PS1dE9 mice. To further understand how ferroptosis participates in the pathogenesis of AD, we also performed gene set enrichment analysis (GSEA) [39, 40] for ferroptosis-related RNA-seq data (GSE126787), which were published recently and showed transcriptional responses to ferroptotic stimuli of wild-type primary cortical neurons in vitro [41] (Fig. S9). The results of this analysis revealed an obvious overrepresentation of upregulated genes in a ferroptosis-related array associated with AD (Supplementary Fig. S9A, *p* < 0.0001, FDR < 0.0001). Eighty-one genes (out of 151 genes in KEGG genes sets of AD (https://www.gsea-msigdb.org/gsea/downloads.jsp) were core enriched in the ferroptosis arrays. Further comparative analyses by Metascape [42] showed that the function of these core enrichment genes were critical for ATP metabolic processes, reactive oxygen species metabolic processes, mitochondrial protein import and amyloid beta formation (Supplementary Fig. S9B). The results of this analysis inferred that ferroptosis could play a role in neuronal loss and impaired cognitive in AD.

### Inhibitors of ferroptosis ameliorated the neuronal death and memory impairment induced by Aβ1–42

**Fig. 5:**
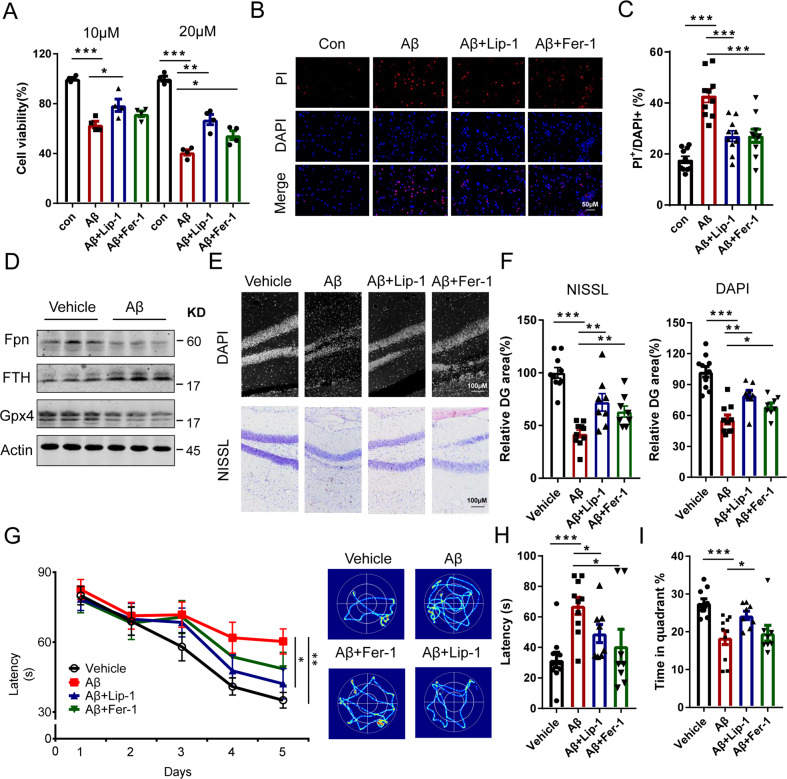
Inhibitors of ferroptosis ameliorated the neuronal death and memory impairment induced by Aβ_1–42_ aggregation in vitro and in vivo. **A** Primary neurons were exposed to 10 μm/20 μm Aβ_1–42_ and ferroptotic inhibitors for 24 h. The cell viability was accessed by CCK8 assays (*n* = 4). **B** After treatment (10 μm Aβ_1–42_, 100 nM Lip-1 or 1 μm Fer-1), the neurons were stained with PI and DAPI. Representative images and **C** percentage of PI + /DAPI + cells are shown (*n* = 10). **D** Protein levels of Fpn, FTH, and Gpx4 in the hippocampus exposed to Aβ1–42. **E** Representative images of Nissl and PI staining of the dentate gyrus of the mice exposed to Aβ_1–42_ and ferroptotic inhibitors. **F** The quantification for **E** (vehicle *n* = 10; Aβ *n* = 10; Aβ + Lip-1 *n* = 8; Aβ + Fer-1 *n* = 8). **G** The latency during the learning stages (left) and the representative searching trace (right) in the Morris water maze of the mice exposed to Aβ_1–42_ injection and ferroptotic inhibitors (vehicle, *n* = 10; Aβ *n* = 10; Aβ + Fer-1 *n* = 8; Aβ + Lip-1 *n* = 8). **H** The latency to reach a hidden platform as well as (I) time in the target quadrant on day 7 of these mice. Data are shown as the mean ± SD of at least three independent experiments. Statistical analyses were carried out using two-way ANOVA and mutiple t-tests. *p < 0.05; **p < 0.01; ***p < 0.001. Detailed Statistical analyses are included with the Supplementary Table S4.

To determine whether ferroptosis is responsible for the neuronal cell death and cognitive impairment induced by Aβ in AD, we treated the primary hippocampal neurons of mice with Aβ1–42 and inhibitors of ferroptosis, liproxstatin1 (Lip-1) or ferrostatin1 (Fer-1). Cell death was assessed with propidium iodide (PI) staining and CCK8. Primary neurons exposed to Aβ1–42 showed significant neuronal death, while treatment with Lip-1 or Fer-1 could partially reduce cell death (Fig. 5A–C). These results suggested that ferroptosis is involved in the neuronal death induced by Aβ in AD in vitro. In previous studies, necrosis and apoptosis were also reported to be involved in Aβ induced neuronal loss [43, 44]. Therefore, we also detected the effects of inhibitors of apoptosis (Emricasan) and necrosis (Nec-1) as controls. The results showed that all three of these inhibitors could partially block Aβ induced neuronal death in vitro, while Lip-1 (a ferroptosis inhibitor) showed the best preventive effects among these three inhibitors (Supplementary Fig. S10 A,B). We also examined the expression levels of Fpn under Aβ treatment. Fpn was downregulated in both the Aβ_1–42_ injected hippocampus (Fig. 5D) and the Aβ_1–42_ treated primary neurons (Supplementary Fig. S10C). Besides, the tissue iron content and ferritin level were elevated, while GPX4 levels were decreased in the hippocampus after exposure to an injection of Aβ, which inferred that Aβ could directly impact on ferroptosis in neurons (Figs. 5D and Supplementary Fig. S11). To further confirm our hypothesis, we also treated the mice with inhibitors of ferroptosis after stereotaxic infusion of oligomeric Aβ1–42 into the hippocampus in vivo. The results showed that administration of both inhibitors after Aβ injection could effectively reduce the neuronal death induced by Aβ injection in the dentate gyrus of the hippocampus (Fig. 5E, F). In addition, liproxstatin treatment could also ameliorate the impairment of learning and memory induced by Aβ exposure (including the learning strategy during the training sessions and the time spent in the target quadrant during the probe trial) on the Morris water maze test (Fig. 5G–I). The effects of ferrostatin were not as apparent as those of liproxstatin, only the latency during the probe trial was improved, while the time spent in the target quadrant was not altered by drug administration (Fig. 5G–I). These results demonstrate the involvement of ferroptosis in the neuronal cell death and memory loss induced by Aβ in AD.

### Elevation of Fpn in the hippocampus ameliorated ferroptosis and memory loss in *APPswe/PS1dE9* mice

**Fig. 6:**
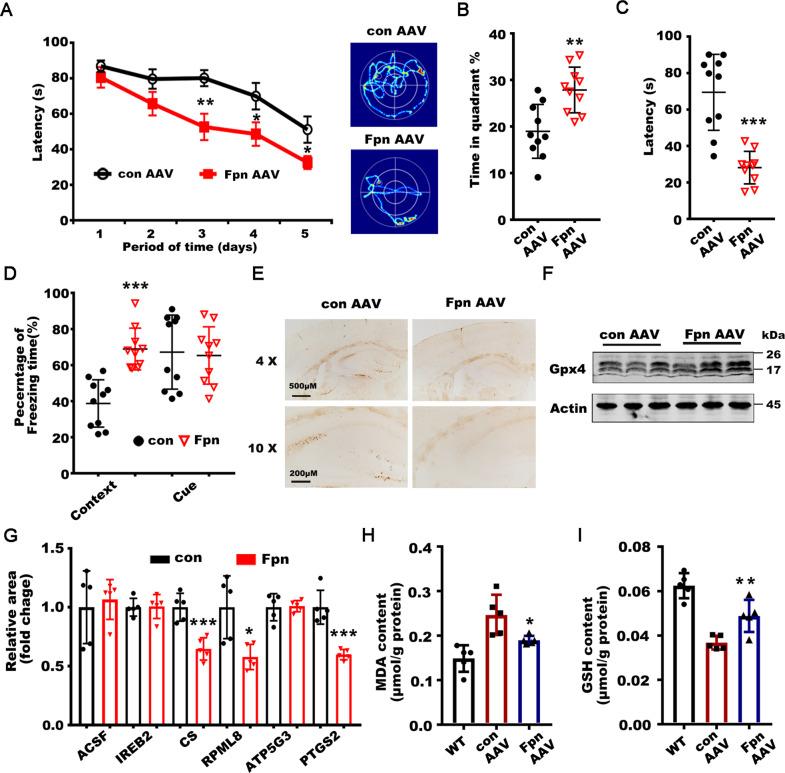
Restoring of Fpn in hippocampus ameliorated ferroptosis and memory loss in *APPswe/PS1dE9* mice. **A** The latency (left) and the representative searching trace (right) to the platform of APPswe/PS1dE9 mice with AAVs overexpressed full length murine Fpn (Fpn-AAV, *n* = 10) or corresponding con-AAVs (con AAV, *n* = 10) injection during the training process at 12 months old. **B** The time in the target quadrant as well as **C** the latency to reach a hidden platform on day 7 of APPswe/PS1dE9 mice with con-AAV or Fpn-AAV injection. **D** Increased time spent freezing in the contextual fear conditioning of the APPswe/PS1dE9 mice with Fpn-AAV injection (*n* = 10). **E** The DAB-enhanced Perl’s Prussian blue iron staining of the hippocampus of the AAV injected APPswe/PS1dE9 mice. **F** The protein levels of Gpx-4 were detected in hippocampus of the APPswe/PS1dE9 mice with con-AAV or Fpn-AAV injection. β-Actin served as a loading control. **G** The mRNA level of ACSF2, IREB2, CS, RPL8, ATPG53 and PTGS2 were detected in the hippocampus of the APPswe/PS1dE9 mice with con-AAV (*n* = 5) or Fpn-AAV(*n* = 5) injection. β-Actin was used as an internal control, and results are shown as fold change of the control. **H** The MDA content and **I** the GSH content in the hippocampus of the APPswe/PS1dE9 mice with con-AAV (*n* = 5) or Fpn-AAV(*n* = 5) injection compared to the wild-type controls. Data are shown as the mean ± SD of at least three independent experiments. Statistical analyses were carried out using two-way ANOVA and mutiple *t*-tests. *p < 0.05; **p < 0.01; ***p < 0.001. Detailed Statistical analyses are included with the Supplementary Table S4.

**Fig. 7:**
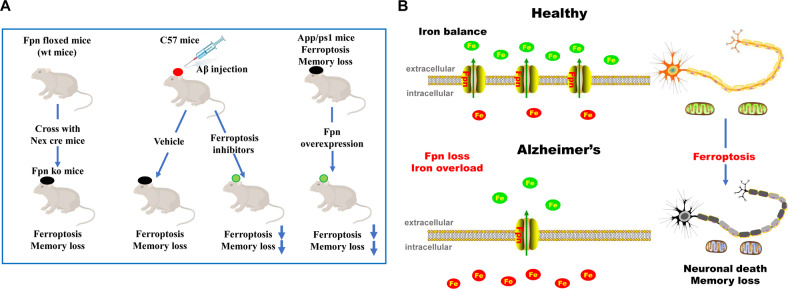
Graphical abstract of memory impairment and ferroptosis induced by Fpn loss in Alzheimer’s disease. **A** A schematic diagram for the mouse models in this study. **B** Graphical abstract of the role of ferroptosis induced by deficiency of Fpn in Alzheimer’s disease.

We finally evaluated whether restoration of the level of Fpn could ameliorate the memory impairment and ferroptosis in AD. To this end, we injected the AAV packaged full length murine Fpn cDNA into the hippocampus of APPswe/PS1dE9 mice at 9 months old (Supplementary Fig. S12A, B). We found that overexpression of Fpn could reduce the memory decline of the AD model mice at 12 months old. The mice with overexpressed Fpn had improved memory performance (the learning strategy during the training sessions and the time spent in the target quadrant during the probe trial) on Morris water maze tests compared with the control mice (Figs. 6A–C and 7). In the fear condition test, overexpression of Fpn also increased the freezing time of AD mice (Fig. 6D). No significant differences in basal locomotive behavior or sensitivity to electrical shocks were observed between the two groups (Supplementary Fig. S12C, D). Furthermore, the abnormal iron accumulation and aberrant expressions of genes related to ferroptosis were partially reversed in the Fpn overexpressed mice (Fig. 6E–G). The MDA levels and GSH content of the hippocampus were also ameliorated by the Fpn-expressed virus injection (Fig. 6H, I). Altogether, these results demonstrated that overexpression of Fpn could partially prevent the memory decline and ameliorated ferroptosis in AD mice (Fig. 7).

## Discussion

Recently, excess deregulated brain iron has been widely reported in the pathogenesis of neurodegenerative diseases such as Parkinson’s disease (PD) [45], Huntington’s disease [46], amyotrophic lateral sclerosis (ALS) [47], and Alzheimer’s disease (AD) [1, 48]. However, the underlying mechanisms and roles of iron regulation associated genes in the disturbance of iron homeostasis remain elusive. Here, we demonstrated that Fpn, the only known iron exporter, was downregulated with age in an AD mouse model and AD patients, consistent with multiple previous reports [25, 49, 50]. Interestingly, the mRNA level of Fpn was not altered in AD, suggested potential posttranscriptional deregulation for Fpn. According to a website based prediction tool, Targetscan, Fpn is a potential target for miR-124, which is a brain-enriched miRNA [51] that is specifically upregulated in the hippocampus and temporal cortex of AD patients [35]. We hypothesize that the loss of Fpn might be partially caused by elevated levels of miR-124 in AD. A previous study also indicated that the Aβ toxicity induced by directly injecting Aβ oligomers into the hippocampus could increase APP levels and iron accumulation, and this process was mediated by tau [52]. In our study, cytotoxicity due to high concentrations of Aβ_1–42_ could directly induce downregulation of Fpn in the primary neurons and hippocampus, indicating a complicated vicious circle of Aβ formation, Fpn downregulation and iron accumulation.

To understand the relationship of Fpn loss with AD-like pathological/behavioral phenotypes, we generated a mouse strain with selective knockout of Fpn in the excitatory neurons of the hippocampus and neocortex and found that Fpn^fl/fl/NEXcre^ mice developed obvious hippocampal atrophy and memory impairments, as seen in AD. Ferroptosis is morphologically, biochemically, and genetically distinct from other forms of cell death. Ferroptosis activation is dependent upon the levels of intracellular iron and can be specifically inhibited using an iron chelator [10]. Recent studies have indicated that ferroptosis contributes to pathological processes in a variety of diseases of the nervous system, including acute organ failure after ischemia/reperfusion [12], Huntington’s disease, and other neurodegenerative diseases [53]. However, little is known about the role of ferroptosis in AD. GSEA of ferroptosis-related RNA-seq data [12] (GSE126787) showed that differentially expressed genes in ferroptosis are highly enriched in the AD gene sets of KEGG. The functions of core enrichment genes are distributed in the pathways of ATP metabolic processes, ROS metabolic processes, mitochondrial protein import and amyloid beta formation, implying the important role of ferroptosis in AD pathogenesis.

Recent studies have revealed the widespread existence of ferroptosis in neurological disorders, including neurodegenerative diseases and brain damage, as a way to promote cell death [12, 13, 15, 17, 54]. Inhibitors of ferroptosis, such as with ferrostatins-1 and liproxstatins-1, could ameliorate neuronal loss and cognitive disorders in models of degenerative brain disorders such Parkinson’s disease, as well as ischemic and hemorrhagic stroke [13, 53–55]. Therefore, optimization and development of inhibitors of ferroptosis could be a potential approach for these neurological diseases. However, the therapeutic effects of ferroptosis inhibitors in AD models remain elusive. In our study, application of liproxstatin-1, a specific inhibitor of ferroptosis, could protect against neuronal cell death and memory loss induced by Aβ treatment both in vivo and in vitro, suggesting that ferroptosis contributes to the neuronal death induced by Aβ. Moreover, overexpression of Fpn partially alleviated memory impairment and ferroptosis in the AD mouse model, further confirming the critical role of Fpn in AD.

In conclusion, our findings provide evidence to show that loss of the iron exporter protein Fpn participates in the neuronal loss and memory impairment in AD. This study also highlights the involvement of ferroptosis in the pathogenesis of this disease. Thus, targeting Fpn or inhibiting ferroptosis could be a promising therapeutic strategy for AD in the future.

### Supplementary information

Supplementary Table 1

Supplementary Table 2

Supplementary Table 3

Supplementary Table 4

Supplementary Table 5

Supplementary Figure legend

Supplementary Fig. 1

Supplementary Fig. 2

Supplementary Fig. 3

Supplementary Fig. 4

Supplementary Fig. 5

Supplementary Fig. 6

Supplementary Fig. 7

Supplementary Fig. 8

Supplementary Fig. 9

Supplementary Fig. 10

Supplementary Fig. 11

Supplementary Fig. 12

Supplementary Fig. 13

Supplementary Fig. 14
