# Correction: Loss of ferroportin induces memory impairment by promoting ferroptosis in Alzheimer’s disease

**DOI:** 10.1038/s41418-024-01290-w

**Published:** 2024-04-04

**Authors:** Wen-Dai Bao, Pei Pang, Xiao-Ting Zhou, Fan Hu, Wan Xiong, Kai Chen, Jing Wang, Fudi Wang, Dong Xie, Ya-Zhuo Hu, Zhi-Tao Han, Hong-Hong Zhang, Wang-Xia Wang, Peter T. Nelson, Jian-Guo Chen, Youming Lu, Heng-Ye Man, Dan Liu, Ling-Qiang Zhu

**Affiliations:** 1 Department of Pathophysiology, Key Lab of Neurological Disorder of Education Ministry, School of Basic Medicine, Tongji Medical College, Huazhong University of Science and Technology, Wuhan, 430030 PR China; 2 The Institute of Brain Research, Collaborative Innovation Center for Brain Science, Huazhong University of Science and Technology, Wuhan, 430030 PR China; 3 Department of Radiology, Union Hospital, Tongji Medical College, Huazhong University of Science and Technology, Wuhan, Hubei PR China; 4 Department of Nutrition, School of Public Health, Zhejiang University, Hangzhou, Zhejiang 310058 PR China; 5 Institute of Nutritional Science, Shanghai Institutes for Biological Sciences, Chinese Academy of Sciences, 320 Yueyang Road, Shanghai, 200031 PR China; 6 Beijing Key Laboratory of Aging and Geriatrics, National Clinical Research Center for Geriatric Disease, Institute of Geriatrics, Chinese PLA General Hospital and Chinese PLA Medical Academy, Beijing, PR China; 7 Sanders Brown Center on Aging, Pathology and Laboratory Medicine, University of Kentucky, Lexington, KY 40536 USA; 8 Department of Biology, Boston University, Boston, MA 02215 USA

Correction to: *Cell Death & Differentiation* 10.1038/s41418-020-00685-9, published online 04 January 2021

In the initial published version of this article, there was an error in the author information. Dan Liu (liudan_echo@mail.hust.edu.cn) is a co-corresponding author. This correction does not affect the description of the results or the conclusions of this work.

The original article has been corrected.

