# Supplementary material for: Loss of ferroportin induces memory impairment by promoting ferroptosis in Alzheimer’s disease
Source: Cell Death Differ. 2021 Jan 4;28(5):1548–62. doi: 10.1038/s41418-020-00685-9 (PMC8166828; doi:10.1038/s41418-020-00685-9)
Supplement: Supplementary file 1 — Supplementary Table 1 [file 41418_2020_685_MOESM1_ESM.docx]

**Supplementary TABLE 1. Patient informations for correlation analysis**

| **Label** | **Braak** | **MMSE** | **Age at death** | **Sex** | **Educ** | **Apoe** |
| --- | --- | --- | --- | --- | --- | --- |
| C1 | 5 | 26 | 91 | 1 | 18 | 3/4 |
| C2 | 5 | 19.5 | 88 | 1 | 16 | 3/4 |
| C3 | 5 | 4 | 83 | 2 | 12 | 2/4 |
| C4 | 5 | 28 | 75 | 1 | 16 | 3/3 |
| C5 | 5 | 30 | 87 | 2 | 12 | 3/3 |
| C6 | 5 | 13 | 91 | 2 | 12 | 3/3 |
| C7 | 5 | 26 | 82 | 2 | 14 | 3/4 |
| C8 | 1 | 21 | 75 | 2 | 16 | 3/3 |
| C9 | 0 | 28 | 86 | 2 | 18 | 3/3 |
| C10 | 1 | 26 | 84 | 2 | 14 | 3/3 |
| C11 | 1 | 29 | 79 | 2 | 12 | 3/3 |
| C12 | 2 | 28 | 78 | 1 | 16 | 3/3 |
| C13 | 2 | 29 | 81 | 1 | 16 | 3/3 |
| C14 | 2 | 30 | 93 | 2 | 20 | 3/3 |
