# Supplementary material for: Loss of ferroportin induces memory impairment by promoting ferroptosis in Alzheimer’s disease
Source: Cell Death Differ. 2021 Jan 4;28(5):1548–62. doi: 10.1038/s41418-020-00685-9 (PMC8166828; doi:10.1038/s41418-020-00685-9)
Supplement: Supplementary file 2 — Supplementary Table 2 [file 41418_2020_685_MOESM2_ESM.docx]

**Supplementary TABLE 2. Antibody information related to experimental procedures, with catalog number/manufacturer, and used dilutions**

| **Antibody** | **Catalog number/manufacturer** | **Dilution** | **Reference** |
| --- | --- | --- | --- |
| Fpn | MTP11-A, alpha-Diagnostics | 1:1000 for wb，  1:100 for ICH | PMID:29599243 |
| Fpn | NBP1-21502, Novus Biologicals | 1:1000 for wb | PMID:23102618 |
| FTH1 | 4393, Cell Signaling Technology | 1:1000 for wb | PMID:28622511 |
| Gpx4 | 14432-1-AP, Proteintech | 1:250 for wb | PMID:28703400 |
| Beta Actin | 66009-1-Ig ,Proteintech | 1:3000 for wb | PMID:24030155 |
