# Supplementary material for: Loss of ferroportin induces memory impairment by promoting ferroptosis in Alzheimer’s disease
Source: Cell Death Differ. 2021 Jan 4;28(5):1548–62. doi: 10.1038/s41418-020-00685-9 (PMC8166828; doi:10.1038/s41418-020-00685-9)
Supplement: Supplementary file 3 — Supplementary Table 3 [file 41418_2020_685_MOESM3_ESM.docx]

**Supplementary TABLE 3. Sequences of oligonucleotide primers**

| **Target gene** | **Species** | **primer sequence** |
| --- | --- | --- |
| Actin forward | hum | TGGCACCCAGCACAATGAA |
| Actin reverse | hum | CTAAGTCATAGTCCGCCTAGAAGCA |
| Fpn forward | hum | CTACTTGGGGAGATCGGATGT |
| Fpn reverse | hum | CTGGGCCACTTTAAGTCTAGC |
| Actin forward | mus | GAGACCTTCAACACCCCAGC |
| Actin reverse | mus | GGAGAGCATAGCCCTCGTAGAT |
| Fpn forward | mus | GTCGGCCAGATTATGACATTTG |
| Fpn reverse | mus | ATTCCAACCGGAAATAAAACC |
| ACSF2 forward | mus | CTTCGGGAGGCTGTGTATCG |
| ACSF2 reverse | mus | CACCATTCCAGAACTGAGAGC |
| ATPG53 forward | mus | TCTGCATCAGTGTTATCTCGGC |
| ATPG53 reverse | mus | CACCAGAACCAGCAACTCCTA |
| IREB2 forward | mus | TTCTGCCTTACTCAATACGGGT |
| IREB2 reverse | mus | AGGGCACTTCAACATTGCTCT |
| CS forward | mus | GGACAATTTTCCAACCAATCTGC |
| CS reverse | mus | TCGGTTCATTCCCTCTGCATA |
| RPL8 forward | mus | AAGGCGCGGGTTCTGTTTT |
| RPL8 reverse | mus | GCTCTGTCCGCTTCTTGAATC |
| PTGS2 forward | mus | TTCAACACACTCTATCACTGGC |
| PTGS2 reverse | mus | AGAAGCGTTTGCGGTACTCAT |
