# Supplementary material for: Loss of ferroportin induces memory impairment by promoting ferroptosis in Alzheimer’s disease
Source: Cell Death Differ. 2021 Jan 4;28(5):1548–62. doi: 10.1038/s41418-020-00685-9 (PMC8166828; doi:10.1038/s41418-020-00685-9)
Supplement: Supplementary file 4 — Supplementary Table 4 [file 41418_2020_685_MOESM4_ESM.docx]

**Supplementary TABLE 4. Detailed statistical analysis information**

| **Figure and nr of animals or cells used** | **Statistical analysis** | | **Post hoc Tukey’s test**  **or**  **mutiple t-test** | **Mean ± SD/SEM** | |
| --- | --- | --- | --- | --- | --- |
| **Figure1B** |  | |  |  | |
| C57 1M (n=3) | Two-way ANOVA | | 1m WT vs APP/PS1 ,P=0.9846 | C57 1M 1.032 ±0.2519 | |
| APP/PS1 1M (n=3) | age, F (4, 20) = 15.1, P<0.0001 | | 3m WT vs APP/PS1 ,P=0.0192 | App/ps1 1M 1.036±0.2768 | |
| C57 3M (n=3) | genotype,F (1, 20) =20.16,P=0.0002 | | 6m WT vs APP/PS1 ,P=0.0461 | C57 3M 1.071±0.1924 | |
| APP/PS1 3M (n=3) | age x genotype，  F (4, 20) = 1.434 ， P=0.2595 | | 9m WT vs APP/PS1 ,P=0.0008 | App/ps1 3M 0.6419±0.03973 | |
| C57 6M (n=3) |  | | 20m WT vs APP/PS1 ,P=0.0070 | C57 6M 0.8452±0.2952 | |
| APP/PS1 6M (n=3) |  | |  | App/ps1 6M 0.5005 ± 0.08375 | |
| C57 9M (n=3) |  | |  | C57 9M 0.5979±0.05359 | |
| APP/PS1 9M (n=3) |  | |  | App/ps1 9M 0.2745±0.03093 | |
| C57 20M (n=3) |  | |  | App/ps1 20M 0.5588±0.08418 | |
| APP/PS1 20M (n=3) |  | |  | App/ps1 20M 0.2642 ±0.05452 | |
| **Figure1E** |  | |  |  | |
| Con (n=9) |  | | WT vs APP/PS1 ,P=0.0455 | CON 0.997 ± 0.5653 | |
| AD (n=10) |  | |  | AD 0.5637 ± 0.2695 | |
| **Figure1F** |  | |  |  | |
| Human sample(n=14) | Linear regression | |  |  | |
| (con,n=7; AD,n=7) | Y = 6.636*X +26.83 | | P=0.0080, R^2^ =0.4563 |  | |
| **Figure2C** |  | |  |  | |
| **hipo weight** |  | |  |  | |
| WT 1M (n=5) | Two-way ANOVA | | 1m WT vs KO ,P=0.0104 | WT 1M 15.85 ±1.188 | |
| Fpn KO 1M (n=5) | age, F (1, 16) = 38.56 ,P<0.0001 | | 3m WT vs KO ,P=0.0112 | Fpn KO 1M 11.846±2.037 | |
| WT 3M (n=5) | genotype,F (1, 16) = 26.39,P<0.0001 | | 1m WT vs 3m WT ,P=0.0024 | WT 3M 20.646 ±1.154 | |
| Fpn KO 3M (n=5) | age x genotype，  F (1, 16) = 0.0006,P=0.9797 | | 1m KO vs 3m KO ,P=0.0022 | Fpn KO 3M 16.682±2.266 | |
| **brain weight** |  | |  |  | |
| WT 1M (n=5) | Two-way ANOVA | | 1m WT vs KO , P=0.0214 | WT 1M 407.928±10.216 | |
| Fpn KO 1M (n=5) | age, F (1, 16) = 104 ,P<0.0001 | | 3m WT vs KO , P=0.0266 | Fpn KO 1M 375.302±24.788 | |
| WT 3M (n=5) | genotype, F (1, 16) = 20.98, P=0.0003 | | 1m WT vs 3m WT ,P<0.0001 | WT 3M 478.810±13.738 | |
| Fpn KO 3M (n=5) | age x genotype，  F (1, 16) = 0.0006, P=0.9386 | | 1m KO vs 3m KO ,P<0.0001 | Fpn KO 3M 447.280±8.559 | |
| **Figure2D** |  | |  |  | |
| HIPO WT (n=4) |  | | lv WT vs KO ,P=0.0254 | LV WT 1±0.139 | |
| HIPO Fpn KO (n=4) |  | | hipo WT vs KO , P=0.0180 | LV Fpn KO 1.540±0.23 | |
| LV WT (n=3) |  | |  | hipo WT 1±0.1567 | |
| LV Fpn KO (n=3) |  | |  | hipo Fpn KO 0.6685 ±0.1332 | |
| **Figure 2F**  WT 3M (n=5)  Fpn KO 3M (n=5) |  | | WT vs KO ,P=0.0075 | WT 1 ± 0.07044  KO 0.8419 ±0.07041 | |
| **Figure 2H** |  | |  |  | |
| **hipo iron content** |  | |  |  | |
| WT 1M (n=4) | Two-way ANOVA | | 1m WT vs KO ,P=0.0106 | WT 1M 8.215±1.573 | |
| Fpn KO 1M (n=4) | age,F (1, 12) = 17.09，P=0.0014 | | 3m WT vs KO ,P=0.0429 | Fpn KO 1M 11.642±1.793 | |
| WT 3M (n=4) | genotype, F (1, 12) = 10.79, P=0.0065 | | 1m WT vs 3m WT ,P=0.0266 | WT 3M 12.574±1.742 | |
| Fpn KO 3M (n=4) | age x genotype，  F (1, 12) = 0.02674，P=0.8728 | | 1m KO vs 3m KO ,P=0.0874 | Fpn KO 3M 16.359±3.249 | |
|  |  | |  |  | |
|  |  | |  |  | |
|  |  | |  |  | |
|  |  | |  |  | |
|  |  | |  |  | |
| **Figure 3A** |  | |  | Fold change | |
| WT 3M (n=6) |  | | Static T, WT vs KO ,P=0.3984 | Static T, WT vs KO  1.134 ±0.3514 | |
| Fpn KO 3M (n=6) |  | | Move T, WT vs KO ,P=0.4166 | Move T, WT vs KO 0.973±0.0725 | |
|  |  | | Move D, WT vs KO ,P=0.8219 | Move D, WT vs KO 1.030±0.2893 | |
|  |  | | Center T, WT vs KO ,P=0.7303 | Center T,WT vs KO 0.903±0.3254 | |
|  |  | | Corner T, WT vs KO ,P=0.0994 | Corner T,WT vs KO 0.898±0.0382 | |
|  |  | | Side T, WT vs KO ,P=0.0258 | Side T, WT vs KO 1.143±0.0899 | |
| **Figure3B** |  | |  |  | |
| **Morris water maze** | Two-way ANOVA | |  | **Mean ± s.e.m.** | |
| WT 8M (n=20) |  | | Day1 WT vs KO ,P=0.8549 | Day1 WT 78.402±2.952 | |
| Fpn KO 8M (n=18) |  | | Day2 WT vs KO ,P=0.0028 | Day2 WT 48.905±4.353 | |
|  |  | | Day3 WT vs KO ,P=0.0002 | Day3 WT 34.405±3.770 | |
|  |  | | Day4 WT vs KO ,P=0.0006 | Day4 WT 30.063±4.519 | |
|  |  | | Day5 WT vs KO ,P=0.0002 | Day5 WT 24.487±3.103 | |
|  |  | |  | Day1 KO 77.165±3.238 | |
|  |  | |  | Day2 KO 69.365±5.486 | |
|  |  | |  | Day3 KO 59.898±5.830 | |
|  |  | |  | Day4 KO 53.540±6.061 | |
|  |  | |  | Day5 KO 49.773±6.716 | |
| **Figure 3C** |  | |  |  | |
| WT 3M (n=20) |  | | WT vs KO ,P=0.8780 | WT 20591±2827 | |
| Fpn KO 3M (n=18) |  | |  | KO 20718±2155 | |
| **Figure3 D,E** |  | |  |  | |
| **Time in quardrant** |  | |  |  | |
| WT 8M (n=20) |  | | WT vs KO ,P<0.0001 | WT 31.2 ± 6.071 | |
| Fpn KO 8M (n=18) |  | |  | KO 20.96 ±2.99 | |
|  |  | |  |  | |
| **Lantency** |  | |  |  | |
| WT 8M (n=20) |  | | WT vs KO ,P<0.0001 | WT 12.9 ± 6.849 | |
| Fpn KO 8M (n=18) |  | |  | KO 40.5 ± 16.4 | |
| **Figure 3F** |  | |  |  | |
| WT 8M (n=20) |  | | context | context | |
| Fpn KO 8M (n=18) |  | | WT vs KO ,P<0.0001 | WT 66.66 ±14.4 | |
|  |  | |  | KO 36.7 ± 12.97 | |
|  |  | | cue | cue | |
|  |  | | WT vs KO ,P=0.7352 | WT 63.51 ± 14.63 | |
|  |  | |  | KO 61.73 ± 18.46 | |
| **Figure 3G** |  | |  |  | |
| WT 8M (n=20) |  | | During training | During training | |
| Fpn KO 8M (n=16-18) |  | | WT vs KO ,P=0.1407 | WT 29.66±9.808 | |
|  |  | | Before training | KO 24.27±12.18 | |
|  |  | | WT vs KO ,P=0.5176 | Before training | |
|  |  | |  | WT 19.39±11.61 | |
|  |  | |  | KO 16.98±10.51 | |
| **Figure 3I** |  | |  |  | |
| Morris water maze | Two-way ANOVA | |  | **Mean ± s.e.m.** | |
| C57 RNAi scramble(n=8) |  | | Day1 con vs Fpn RNAi,P=0.6431 | Day1 con 81.766 ± 4.004 | |
| C57 RNAi Fpn (n=10) |  | | Day2 con vs Fpn RNAi,P=0.4439 | Day2 con 79 .675 ± 3.929 | |
|  |  | | Day3 con vs Fpn RNAi,P=0.5918 | Day3 con 62.825 ± 5.548 | |
|  |  | | Day4 con vs Fpn RNAi,P=0.0018 | Day4 con 49.728 ± 6.812 | |
|  |  | | Day5con vs Fpn RNAi, P=0.0218 | Day5 con 41.290 ± 6.139 | |
|  |  | |  | Day1 Fpn RNAi  85.172 ± 2.943 | |
|  |  | |  | Day2 Fpn RNAi  74.044 ± 5.487 | |
|  |  | |  | Day3 Fpn RNAi  66.766 ± 5.477 | |
|  |  | |  | Day4 Fpn RNAi  74.953 ± 5.908 | |
|  |  | |  | Day5 Fpn RNAi  59.241 ± 6.272 | |
| **Figure 3K,L** |  | |  |  | |
| **Time in quardrant** |  | |  |  | |
| C57 RNAi scramble (n=8) |  | | con vs Fpn RNAi,P=0.0061 | CON 26.18 ± 5.116 | |
| C57 RNAi Fpn (n=10) |  | |  | Fpn RNAi 16.41 ± 7.43 | |
| **Lantency** |  | |  |  | |
| C57 RNAi scramble (n=8) |  | | con vs Fpn RNAi,P=0.0472 | CON 23.3 ± 12.36 | |
| C57 RNAi Fpn (n=10) |  | |  | Fpn RNAi 49.33 ± 12.78 | |
| **Figure 3J** |  | |  |  | |
| C57 RNAi scramble(n=8) |  | | con vs Fpn RNAi ,P=0.8253 | CON 15553 ± 2627 | |
| C57 RNAi Fpn (n=10) |  | |  | Fpn RNAi 15712 ± 1754 | |
| **Figure 4C** |  | |  |  | |
| MDA CONTENT |  | |  | Fpn f/f 0.1447 ± 0.01436 | |
| Fpn f/f (n=5) |  | | KO vs f/f, P=0.0007 | Fpn KO 0.2761 ± 0.05257 | |
| Fpn KO (n=5) |  | | WT vs APP/PS1 , P=0.0035 | WT 0.1564 ± 0.03436 | |
| WT (n=5) |  | |  | APP/PS1 0.246 ± 0.03514 | |
| APP/PS1(n=5) |  | |  |  | |
| **Figure 4D** |  | |  |  | |
| GSH CONTENT |  | |  |  | |
| Fpn f/f (n=5) |  | | KO vs f/f, P<0.0001 | Fpn f/f 0.074 ± 0.001884 | |
| Fpn KO (n=5) |  | | WT vs APP/PS1 , P=0.0005 | Fpn KO 0.03606 ± 0.004187 | |
| WT (n=5) |  | |  | WT 0.07207 ± 0.002555 | |
| APP/PS1(n=5) |  | |  | APP/PS1 0.04182 ± 0.01168 | |
| **Figure 4G** |  | |  | Fold change | |
| WT 8M (n=5) |  | | ACSF, WT vs KO ,P=0.2459 | ACSF 1.167±0.2789 | |
| Fpn KO 8M (n=5) |  | | IREB2, WT vs KO ,P<0.0001 | IREB2 1.274±0.0582 | |
|  |  | | CS, WT vs KO ,P=0.0031 | CS 1.269±0.1338 | |
|  |  | | RPML8, WT vs KO ,P<0.0001 | RPL8 1.330±0.04677 | |
|  |  | | ATP5G3,WT vs KO ,P=0.0137 | ATP5G3 1.301±0.179 | |
|  |  | | PTSG2, WT vs KO ,P=0.0171 | PTSG2 1.466±0.3397 | |
| **Figure 4H** |  | |  | Fold change | |
| C57 8M (n=5) |  | | ACSF,C57 vsApp/ps1,P<0.0001 | ACSF 2.323±0.3663 | |
| App/ps1 8M (n=5) |  | | IREB2, C57 vsApp/ps1,P<0.0001 | IREB2 1.945±0.2517 | |
|  |  | | CS, C57 vs App/ps1, P=0.0001 | CS 1.843±0.2494 | |
|  |  | | RPML8, C57vsApp/ps1,P=0.0049 | RPL8 1.517±0.2952 | |
|  |  | | ATP5G3,C57vsApp/ps1,  P=0.6697 | ATP5G3 1.096±0.4383 | |
|  |  | | PTSG2,C57 vsApp/ps1,P=0.0008 | PTSG2 1.3443±0.0796 | |
| **Figure 5A** |  | |  |  | |
| **CCK8 caculation**  10μM |  | |  |  | |
| con (n=4) |  | | con vs Aβ, P<0.0001 | con 100±2.102 | |
| Aβ (n=4) |  | | Aβ vs Aβ +Lip-1 , P=0.0335 | Aβ 63.3±5.355 | |
| Aβ +Lip-1 (n=4) |  | | Aβ vs Aβ + Fer-1 , P=0.0522 | Aβ +Lip-1 78.72±9.882 | |
| Aβ + Fer-1 (n=4) |  | |  | Aβ + Fer-1 71.87±4.655 | |
| 20μM |  | |  |  | |
| Aβ (n=4) |  | | con vs Aβ, P<0.0001 | con 100±4.052 | |
| Aβ +Lip-1 (n=4) |  | | Aβ vs Aβ +Lip-1 , P=0.0013 | Aβ 40.93±4.268 | |
| Aβ + Fer-1 (n=4) |  | | Aβ vs Aβ + Fer-1 , P=0.0155 | Aβ +Lip-1 67.26±8.226 | |
| WT Lip-1 (n=4) |  | |  | Aβ + Fer-1 54.64±6.992 | |
| **Figure 5C** |  | |  |  | |
| **PI caculation** |  | |  |  | |
| con (n=10) |  | | con vs Aβ, P<0.0001 | con 17.72±4.445 | |
| Aβ (n=10) |  | | Aβ vs Aβ +Lip-1 , P=0.0002 | Aβ 42.89±8.468 | |
| Aβ +Lip-1 (n=10) |  | | Aβ vs Aβ + Fer-1 , P=0.0005 | Aβ +Lip-1 27.05±6.631 | |
| Aβ + Fer-1 (n=10) |  | |  | Aβ + Fer-1 27.14±8.144 | |
| **Figure 5F** | |  |  | |  |
| **Nissl caculation** | |  |  | |  |
| con (n=10) | |  | con vs Aβ, P<0.0001 | | con 100±15.92 |
| Aβ (n=10) | |  | Aβ vs Aβ +Lip-1 , P=0.0026 | | Aβ 42.12±11.33 |
| Aβ +Lip-1 (n=8) | |  | Aβ vs Aβ + Fer-1 , P=0.0036 | | Aβ +Lip-1 72.02±23.42 |
| Aβ + Fer-1 (n=8) | |  |  | | Aβ + Fer-1 63.26±15.07 |
| **DAPI caculation** | |  |  | |  |
| con (n=10) | |  | con vs Aβ, P<0.0001 | | con 102.4±16.29 |
| Aβ (n=10) | |  | Aβ vs Aβ +Lip-1 , P=0.0020 | | Aβ 56.13±13.79 |
| Aβ +Lip-1 (n=8) | |  | Aβ vs Aβ + Fer-1 , P=0.0405 | | Aβ +Lip-1 79.67±13.05 |
| Aβ + Fer-1 (n=8) | |  |  | | Aβ + Fer-1 68.9±9.442 |
| **Figure 5G** | |  | Mean ± s.e.m. | | **Mean ± s.e.m.** |
| **Morris water maze** | | Two-way ANOVA | Day1 con 79.985±3.967 | | Day1Aβ +Lip-1 78.46±4.913 |
| con (n=10) | | Aβ vs vehicle, P=0.0001  Aβ +Lip-1 vs Aβ ,P=0.0296  Aβ + Fer-1 vs Aβ, P=0.1547 | Day2 con 68.945±6.029 | | Day2 Aβ +Lip-1 69.83±6.792 |
| Aβ (n=10) | |  | Day3 con 57.986±6.072 | | Day3 Aβ +Lip-1 68.44±6.172 |
| Aβ +Lip-1 (n=8) | |  | Day4 con 40.925±3.674 | | Day4 Aβ +Lip-1 47.75±6.679 |
| Aβ + Fer-1 (n=8) | |  | Day5 con 35.105±7.417 | | Day5 Aβ +Lip-1 42.14±6.520 |
|  | |  | Day1 Aβ 82.56±4.321 | | Day1 Aβ + Fer-1 78.04±5.514 |
|  | |  | Day2 Aβ71.31±5.755 | | Day2 Aβ + Fer-1 68.18±7.047 |
|  | |  | Day3 Aβ 71.74±5.411 | | Day3 Aβ + Fer-1 70.74±7.057 |
|  | |  | Day4 Aβ 61.89±6.678 | | Day4 Aβ + Fer-1 53.68±9.014 |
|  | |  | Day5 Aβ 60.31±5.375 | | Day5 Aβ + Fer-1 48.53±7.071 |
| **Figure 5 H.I** | |  |  | |  |
| **Lantency** | |  |  | |  |
| con (n=10) | |  | con vs Aβ, P=0.0002 | | con 31.82±16.1 |
| Aβ (n=10) | |  | Aβ vs Aβ +Lip-1 , P=0.0430 | | Aβ 67.27±17.84 |
| Aβ +Lip-1 (n=8) | |  | Aβ vs Aβ + Fer-1 , P=0.0377 | | Aβ +Lip-1 49.14±16.79 |
| Aβ + Fer-1 (n=8) | |  |  | | Aβ + Fer-1 40.9±31.09 |
| **Time in quardrant** | |  |  | |  |
| con (n=10) | |  | con vs Aβ, P=0.0004 | | con 27.61±3.482 |
| Aβ (n=10) | |  | Aβ vs Aβ +Lip-1 , P=0.0206 | | Aβ 18.46±5.788 |
| Aβ +Lip-1 (n=8) | |  | Aβ vs Aβ + Fer-1 , P=0.6809 | | Aβ +Lip-1 24.33±3.145 |
| Aβ + Fer-1 (n=8) | |  |  | | Aβ + Fer-1 19.63±5.96 |
|  | |  |  | |  |
| **Figure6A** | |  |  | |  |
| **Morris water maze** | | Two-way ANOVA |  | | **Mean ± s.e.m.** |
| CON AAV App/ps1 (n=10) | |  | Day1 con vs Fpn,P=0.4533 | | Day1 con 86.816±3.238 |
| FPN AAV App/ps1 (n=10) | |  | Day2 con vs Fpn,P=0.1040 | | Day2 con 79.627±5.492 |
|  | |  | Day3 con vs Fpn,P=0.0020 | | Day3 con 80.091±4.529 |
|  | |  | Day4 con vs Fpn,P=0.0159 | | Day4 con 69.909±7.482 |
|  | |  | Day5 con vs Fpn,P=0.0358 | | Day5 con 51.125±7.417 |
|  | |  |  | | Day1 Fpn 80.558±5.787 |
|  | |  |  | | Day2 Fpn 65.683±6.640 |
|  | |  |  | | Day3 Fpn 52.618±7.467 |
|  | |  |  | | Day4 Fpn 48.618±6.581 |
|  | |  |  | | Day5 Fpn 32.570±3.680 |
| **Figure 6B,C** | |  |  | |  |
| **Time in quardrant** | |  |  | |  |
| CON AAV App/ps1 (n=10) | |  | CON vs Fpn,P=0.0016 | | CON 18.97±5.776 |
| FPN AAV App/ps1 (n=10) | |  |  | | Fpn 27.85±4.903 |
| **Lantency** | |  |  | |  |
| CON AAV App/ps1 (n=10) | |  | CON vs Fpn,P<0.0001 | | CON 69.43±20.85 |
| FPN AAV App/ps1 (n=10) | |  |  | | Fpn 28.13 ± 8.937 |
| **Figure 6D** | |  | context | | context |
| CON AAV App/ps1 (n=10) | |  | CON vs Fpn ,P<0.0001 | | CON 38.73±13.1 |
| FPN AAV App/ps1 (n=10) | |  |  | | Fpn 68.91±11.58 |
|  | |  | cue | | cue |
|  | |  | CON vs Fpn ,P=0.8228 | | CON 67.19±20.54 |
|  | |  |  | | Fpn 65.33±15.9 |
| **Figure 6G** | |  |  | | Fold change |
| CON AAV App/ps1 (n=5) | |  | ACSF, con vs Fpn ,P=0.6863 | | ACSF 1.066±0.169 |
| FPN AAV App/ps1 (n=5) | |  | IREB2,con vs Fpn ,P=0.9133 | | IREB2 1.006±0.1022 |
|  | |  | CS,con vs Fpn , P=0.0008 | | CS 0.6464 ±0.09443 |
|  | |  | RPML8, con vs Fpn , P=0.0109 | | RPL8 0.5779±0.1066 |
|  | |  | ATP5G3, con vs Fpn ,P=0.8718 | | ATP5G3 1.009±0.04781 |
|  | |  | PTSG2, con vs Fpn ,P=0.0003 | | PTSG2 0.5980±0.04377 |
|  | |  |  | |  |
| **Figure 6H** |  | |  |  | |
| MDA CONTENT |  | |  |  | |
| WT (n=5) |  | | Con aav vs Fpn aav , P=0.0251 | WT 0.1487±0.02982 | |
| APP/PS1con aav(n=5) |  | |  | Con aav 0.2466±0.0451 | |
| APP/PS1Fpn aav(n=5) |  | |  | Fpn aav 0.19±0.00948 | |
| **Figure 6I** |  | |  |  | |
| GSH CONTENT |  | |  | WT 0.06251±0.005689 | |
| WT (n=5) |  | | Con aav vs Fpn aav , P=0.0093 | APP/PS1 0.03683 ± 0.003137 | |
| APP/PS1con aav(n=5) |  | |  | Fpn aav 0.04886 ± 0.007255 | |
| APP/PS1Fpn aav(n=5) |  | |  |  | |

| **Figure and nr of animals or cells used** | | **Statistical analysis** | **Post hoc Tukey’s test**  **or**  **mutiple t-test** | **Mean ± SD/SEM** | |
| --- | --- | --- | --- | --- | --- |
| **Suppelementary** | |  |  |  | |
| **Figure S1 B,C** | |  |  |  | |
| **Cortex** | |  |  |  | |
| C57 3M (n=9) | |  |  |  | |
| APP/PS1 3M (n=9) | |  | 3m WT vs APP/PS1 , P=0.4075 | 3M C57 vs APP/PS1 0.9211±0.1206 | |
| C57 6M (n=9) | |  | 6m WT vs APP/PS1 , P=0.4900 | 6M C57 vs APP/PS1 0.9097±0.1261 | |
| APP/PS1 6M (n=9) | |  | 20m WT vsAPP/PS1 ,P=0.2270 | 20M C57 vs APP/PS1 0.7032±0.5114 | |
| C57 20M (n=6) | |  |  |  | |
| APP/PS1 20M (n=6) | |  |  |  | |
| **hipo** | |  |  |  | |
| C57 3M (n=9) | |  |  |  | |
| APP/PS1 3M (n=9) | |  | 3m,WT vs APP/PS1 ,P=0.5859 | 3M APP/PS1 C57 0.926±0.188 | |
| C57 6M (n=9) | |  | 6m,WT vs APP/PS1 ,P=0.9074 | 6M APP/PS1 C57 1.011±0.178 | |
| APP/PS1 6M (n=9) | |  | 20m ,WT vs APP/PS1 , P=0.0207 | 20M APP/PS1 C57 1.208±0.041 | |
| C57 20M (n=6) | |  |  |  | |
| APP/PS1 20M (n=3) | |  |  |  | |
| **Figure S1D** | |  |  |  | |
| Con (n=4) | |  | WT vs APP/PS1 ,P=0.1861 | CON 1.21 ± 0.2822 | |
| AD (n=4) | |  |  | AD 0.8433 ± 0.4018 | |
| **Figure S2 B**  C57 1M (n=3)  APP/PS1 1M (n=3)  C57 3M (n=3)  APP/PS1 3M (n=3)  C57 6M (n=3)  APP/PS1 6M (n=3)  C57 9M (n=3)  APP/PS1 9M (n=3)  C57 20M (n=3)  APP/PS120M(n=3) | | Two-way ANOVA  age, F (4, 20) =51.36, P<0.0001  genotype,F (1, 20) =44.5,P<0.0001  age x genotype，  F (4, 20) = 7.296 ， P=0.0009 | 1m WT vs APP/PS1 ,P=0.9980  3m WT vs APP/PS1 ,P=0.9999  6m WT vs APP/PS1 ,P=0.0008  9m WT vs APP/PS1 ,P=0.0066  20m WT vs APP/PS1 ,P<0.0001 | C57 1M 1 ±0.381  App/ps1 1M 1.068±0.227  C57 3M 0.861±0.322  App/ps1 3M 0.861±0.290  C57 6M 0.721±0.179  App/ps1 6M 1.559 ± 0.046  C57 9M 1.698±0.030  App/ps1 9M 2.373±0.113  App/ps1 20M 1.744±0.161  App/ps1 20M 2.863 ±0.184 | |
| **Figure S2 C**  C57 1M (n=3)  APP/PS1 1M (n=3)  C57 3M (n=3)  APP/PS1 3M (n=3)  C57 6M (n=3)  APP/PS1 6M (n=3)  C57 9M (n=3)  APP/PS1 9M (n=3)  C57 20M (n=3)  APP/PS120M(n=3) | | Two-way ANOVA  age, F (4, 20) =27.85, P<0.0001  genotype,F (1, 20) =121.5,P<0.0001  age x genotype，  F (4, 20) = 32.64 ， P<0.0001 | 1m WT vs APP/PS1 ,P=0.5557  3m WT vs APP/PS1 ,P=0.9964  6m WT vs APP/PS1 ,P<0.0001  9m WT vs APP/PS1 ,P=0.0001  20m WT vs APP/PS1 ,P<0.0001 | C57 1M 1 ±0.099  App/ps1 1M 1.088±0.083  C57 3M 0.783±0.049  App/ps1 3M 0.758±0.077  C57 6M 0.901±0.123  App/ps1 6M 0.466 ± 0.021  C57 9M 0.835±0.077  App/ps1 9M 0.513±0.023  App/ps1 20M 1.080±0.062  App/ps1 20M 0.326 ±0.027 | |
| **Figure S2 D**  C57 1M (n=3)  APP/PS1 1M (n=3)  C57 3M (n=3)  APP/PS1 3M (n=3)  C57 6M (n=3)  APP/PS1 6M (n=3)  C57 9M (n=3)  APP/PS1 9M (n=3)  C57 20M (n=3)  APP/PS120M(n=3) | | Two-way ANOVA  age, F (4, 20) =97.58, P<0.0001  genotype,F (1, 20) =55.68,P<0.0001  age x genotype，  F (4, 20) = 3.14 ， P=0.0371 | 1m WT vs APP/PS1 ,P=0.4623  3m WT vs APP/PS1 ,P=0.0343  6m WT vs APP/PS1 ,P=0.0150  9m WT vs APP/PS1 ,P=0.0011  20m WT vs APP/PS1 ,P=0.0132 | C57 1M 9.931 ±0.778  App/ps1 1M 11.151±2.482  C57 3M 14.901±1.328  App/ps1 3M 18.891±1.739  C57 6M 15.946±1.327  App/ps1 6M 23.275± 2.808  C57 9M 21.558±0.509  App/ps1 9M 26.831±0.958  App/ps1 20M 24.803±0.686  App/ps1 20M 28.259 ±1.232 | |
| **Figure S4B** | |  |  |  |  |
| WT 1M (n=3) | | Two-way ANOVA |  | WT 1M 12.913±0.8237 |  |
| Fpn KO 1M (n=3) | | age,F (1, 8) = 94.53，P<0.0001 | 1m WT vs KO ,P=0.7262 | Fpn KO 1M 12.32±2.052 |  |
| WT 3M (n=3) | | genotype, F (1, 8) = 2.106，P=0.1848 | 3m WT vs KO ,P=0.1296 | WT 3M 25.243±1.635 |  |
| Fpn KO 3M (n=3) | | age x genotype，  F (1, 8) = 0.88, P=0.3756 |  | Fpn KO 3M 22.48±2.914 |  |
| **Figure S5B** | |  |  | Fold change | |
| HIPO WT (n=3) | |  | HIPO WT vs KO ,P=0.0286 | HIPO WT 1 ± 0.05978 | |
| HIPO Fpn KO (n=3) | |  | LV WT vs KO , P=0.0382 | HIPO Fpn KO 1.589 ± 0.381 | |
| LV WT (n=3) | |  |  | LV WT 1 ± 0.2471 | |
| LV Fpn KO (n=3) | |  |  | LV Fpn KO 0.532 ± 0.09879 | |
| **FigureS 6 A** |  |  |  | |  |
| WT 1M (n=6) | Two-way ANOVA | 1m WT vs KO , P=0.0108 | WT 1M 5.787±0.9877 | |  |
| Fpn KO 1M (n= 6) | age, F (1, 20) = 21.07,P=0.0002 | 3m WT vs KO , P=0.0098 | Fpn KO 1M 7.729±1。158 | |  |
| WT 3M (n=6) | genotype, F (1, 20) = 16.74,P=0.0006 | 1m WT vs 3m WT , P=0.069 | WT 3M 8.160±2.678 | |  |
| Fpn KO 3M (n=6) | age x genotype，  F (1, 20) = 3.407, P=0.0798 | 1m KO vs 3m KO ,P=0.0014 | Fpn KO 3M 13.295±2.908 | |  |
| **FigureS 6 B,C** | |  |  | Fold change | |
| WT 1M (n=3) | |  | cere, WT vs KO ,P=0.9790 | cere, WT vs KO  0.8275 ± 0.2895 | |
| Fpn KO 1M (n=3) | |  | Liver, WT vs KO ,P=0.6591 | Liver, WT vs KO  0.8862 ± 0.3606 | |
|  | |  | spleen, WT vs KO ,P=0.2946 | spleen, WT vs KO  1.153 ± 0.1523 | |
|  | |  |  | Fold change | |
| WT 3M (n=3) | |  | cere, WT vs KO ,P=0.9595 | cere, WT vs KO  1.008 ± 0.245 | |
| Fpn KO 3M (n=3) | |  | Liver, WT vs KO ,P=0.2801 | Liver,WT vs KO  1.129 ± 0.1373 | |
|  | |  | spleen, WT vs KO ,P=0.5784 | spleen, WT vs KO  1.115 ± 0.187 | |
| **Figure S7B**  CON (n=8)  RNAi (n=10) | |  | con vs RNAi ,P=0.0001 | Con 1 ± 0.07837  RNA i 0.5891 ± 0.04062 | |
| **Figure S10A**  Con (n=5)  Emric (n=5)  Nec1 (n=5)  Lip-1 (n=5) | |  | Con ys Emric, P=0.3101  Con ys Nec-1, P=0.7841  Con ys Lip-1, P=0.5563 | Fold change  Con ys Emric,  1.074 ± 0.04545  Con ys Nec-1,  1.022 ± 0.05828  Con ys Lip-1,  1.046 ± 0.05536 | |
| **Figure S10B**  Con (n=5)  Aβ (n=5)  Aβ+Emric (n=5)  Aβ+Nec-1 (n=5)  Aβ+Lip-1 (n=5) | |  | Con ys Aβ, P<0.0001  Con ys Aβ+Emric, P=0.0073  Con ys Aβ+Nec-1, P=0.0006  Con ys Aβ+Lip-1, P=0.0003 | Fold change  Con ys Aβ, 0.4499 ± 0.0106  Con ys Aβ+Emric,  0.6288 ± 0.04894  Con ys Aβ+Nec-1,  0.6071 ± 0.02636  Con ys Aβ+Lip-1,  0.7202 ± 0.04242 | |
| **Figure S10C**  CON (n=3)  Aβ (n=3) | |  | Con ys Aβ, P=0.0496 | Con, 1 ± 0.1334  Aβ, 0.5261 ± 0.1057, | |
| **Figure S11A**  CON (n=4)  Aβ (n=4) | |  | Con ys Aβ, P=0.0223 | Con 12.87 ± 0.4412  Aβ 16.05 ± 0.9406 | |
| **Figure S11B**  CON (n=3)  Aβ (n=3) | |  | Con ys Aβ  Fpn, P=0.0052  Fth, P=0.0032  Gpx4, P=0.0016 | Fold change  Fpn, 0.609 ± 0.0232  Fth, 1.944 ± 0.1157  Gpx4, 0.6868 ± 0.01516 | |
| **Figure S12C** | |  |  |  | |
| CON AAV App/ps1 (n=10) | |  | con vs Fpn ,P=0.8031 | CON 10292 ± 2863 | |
| FPN AAV App/ps1 (n=10) | |  |  | Fpn 10610± 2751 | |
| **Figure S12D** | |  | During training | During training | |
| CON AAV App/ps1 (n=10) | |  | CON vs Fpn ,P=0.3686 | CON 32.56 ± 12.33 | |
| FPN AAV App/ps1 (n=10) | |  | Before tone | Fpn 27.91 ± 10.11 | |
|  | |  | con vs Fpn ,P=0.7851 | Before tone | |
|  | |  |  | CON 26.65 ±12.6 | |
|  | |  |  | Fpn 25.01 ± 13.85 | |
