# Supplementary material for: Loss of ferroportin induces memory impairment by promoting ferroptosis in Alzheimer’s disease
Source: Cell Death Differ. 2021 Jan 4;28(5):1548–62. doi: 10.1038/s41418-020-00685-9 (PMC8166828; doi:10.1038/s41418-020-00685-9)
Supplement: Supplementary file 5 — Supplementary Table 5 [file 41418_2020_685_MOESM5_ESM.docx]

**Supplementary TABLE 5. Serum parameters of Fpn^fl/fl/NEXcre^ mice and age-matched floxed littermates (Fpn^fl/fl^)**

| **Serum Parameters** |  | **Fpn^fl/fl^** | **Fpn^fl/fl/NEXcre^** | ***P*** |
| --- | --- | --- | --- | --- |
| serum iron | (μg/dL) | 197.22±28.33 | 197.79±29.57 | 0.9806 |
| UIBC | (μg/dL) | 233.26±25.31 | 249.37±38.73 | 0.6983 |
| TIBC | (μg/dL) | 430.48±12.38 | 447.15±77.15 | 0.7847 |
| TS | % | 45.8±6.2 | 44.2±17.1 | 0.9858 |

Three-month-old Fpnfl/fl (n=5) and *Fpn^fl/fl/NEXcre^* mice (n=5) were sacrificed and blood was collected for analysis. Serum iron and Total Iron binding capacity (TIBC) were measured. Unsaturated Iron-Binding Capacity (UIBC) and Transferrin Saturation (TS) were caculated from the relative data. Data are given as the mean ± SEM. Sample P represents the value of a Student's t-test (unpaired, two-tailed)
