# Supplementary material for: Loss of ferroportin induces memory impairment by promoting ferroptosis in Alzheimer’s disease
Source: Cell Death Differ. 2021 Jan 4;28(5):1548–62. doi: 10.1038/s41418-020-00685-9 (PMC8166828; doi:10.1038/s41418-020-00685-9)
Supplement: Supplementary file 6 — Supplementary Figure legend [file 41418_2020_685_MOESM6_ESM.docx]

**Supplemental Figure legend**

**Supplementary Figure 1. Protein level of Fpn was downregulated in the cortex in AD**

(A)The protein level of Fpn in the cortex of APP/PS1 mice at different age (M: month) and the age-matched wild-type littermates. (B) mRNA relative expression level of Fpn in the cortex (C57 1M n=9, 6M n=9, 20M n=6; App/ps1 1M n=9, 6M n=9, 20M n=6) and (C) hippocampus (C57 1M n=9, 6M n=9, 20M n=6; App/ps1 1M n=9, 6M n=9, 20M n=3) in APP/PS1 mice and the age-matched wild-type littermates at different ages. (D) The protein level of Fpn in brain tissues of AD patients and control (CON) sample (temporal pole, 7 con vs 7 AD). (E)The mRNA level of Fpn in tissues of AD patients (n=4). β-Actin was used as an internal control, and results are shown as fold change of the control.

Data are shown as the mean ± SD of at least 3 independent experiments. Statistical analyses were carried out using two-way ANOVA and mutiple t-tests. *p < 0.05; **p < 0.01; ***p < 0.001

**Supplementary Figure 2. Excess iron was accumulated with age in AD mice**

(A) Representive protein level of FTH (Ferritin heavy chain) and Gpx4 in the hippocampus of APPswe/PS1dE9 (APP/PS1) mice at different ages (M: month) and the age-matched wild-type littermates (WT). (B) The quantification for protein level of FTH and (C) Gpx4 in the hippocampus of APPswe/PS1dE9 (APP/PS1) mice (n=3). (D) The tissue iron content (μg/g) in the hippocampus of APPswe/PS1dE9 (APP/PS1) mice at different ages (M: month) and the age-matched wild-type littermates (WT) (n=3).

Data are shown as the mean ± SD of at least 3 independent experiments. Statistical analyses were carried out using two-way ANOVA and mutiple t-tests. *p < 0.05; **p < 0.01; ***p < 0.001

**Supplementary Figure 3. Identification of the Fpn^fl/fl/NEXcre^ mice**

(A) Genotyping PCR on genomic DNA from mouse tails, obtained after intercrossing Fpnfl/fl mice and NEX cre mice. The sizes of PCR products are indicated. (B) PCR analysis of tissue genomic DNA from a Fpn^fl/fl/NEXcre^ mouse. Liver(L), Spleen(S), Kidney(K), Intestine(I), lung (Lu), heart(H), Tail(T), Cerebellum (CE), Cortex(CO), Hippocampus(HI), Strium(ST). The floxed allele is 522 bp, and the full deletion allele is 398 bp. (C) The protein level of Fpn in the primary neurons of cortex and liver tissues of Fpn^fl/fl/NEXcre^ mice and age-matched wild type littermates. β-Actin was served as a loading control.

**Supplementary Figure 4. The whole body weights of Fpn^fl/fl/NEXcre^ mice were not altered compared to littermates.**

(A) Representative images of Fpn^fl/fl/NEXcre^ mice and age-matched wild type littermates. (B) The body weight of Fpn^fl/fl/NEXcre^ mice (n=3) and age-matched wild type littermates (n=3) at 1 month and 3 months of age.

Data are shown as the mean ± SD of at least 3 independent experiments. Statistical analyses were carried out using by two-way ANOVA and mutiple t-test. *p < 0.05; **p < 0.01; ***p < 0.001

**Supplementary Figure 5. Brain atrophy became worsen in Fpn^fl/fl/NEXcre^ mice at 3 months of age.**

(A)The representative MRI images from the brains of Fpn^fl/fl/NEXcre^ mice and age-matched wild type littermates at 3 months old. (B) Quantitative fold change of the relative area of the lateral ventrical (LV) and hippocampus (Hipo) in MRI images (n=3).

Data are shown as the mean ± SD of at least 3 independent experiments. Statistical analyses were carried out using mutiple t-tests. *p < 0.05; **p < 0.01; ***p < 0.001

**Supplementary Figure 6. The tissue iron content of non-targeted organs in** **Fpn^fl/fl/NEXcre^ mice were not altered.**

(A) The tissue iron content (μg/g) in the cortex (n=6) of Fpn^fl/fl/NEXcre^ mice at 1 month old and 3 months old. (B) Tissue iron content of the cerebellum, liver and spleen of Fpn^fl/fl/NEXcre^ mice (n=3) and age-matched wild type littermates (n=3) at 1 month and (C) 3 months of age.

Data are shown as the mean ± SD of at least 3 independent experiments. Statistical analyses were carried out using mutiple t-tests. *p < 0.05; **p < 0.01; ***p < 0.001

**Supplementary Figure 7. The injection of lenti-virus packaged with a shRNA directed against Fpn effectively reduced the Fpn expression**

(A) The Fpn protein level in hippocampus of C57 mice injected with lentivirus expressing shRNA against fpn (FPN-RNAi) or scrambled hairpin (Con virus). (B) The quantitative data of Fpn protein level in hippocampus of C57 mice injected with lentivirus (con, n=8; RNAi, n=10).

Data are shown as the mean ± SD of at least 3 independent experiments. Statistical analyses were carried out using t-tests. *p < 0.05; **p < 0.01; ***p < 0.001

**Supplementary Figure 8 Transmission electron microscopy of hippocampus of Fpn^fl/fl/NEXcre^ and *APP/PS1* mice**

(A)Transmission electron microscopy pictures of cytoplasmic compartment of the hippocampus neurons in Fpnfl/fl, Fpn^fl/fl/NEXcre^, WT, APP/PS1 mice at 9-month-old. n=10 cells from 3 mice per strain. (B) Mitochondrial area frequency in cytoplasmic compartment of these mice. Calculated from n>100 mitochondria from at least 3 mice per group.

**Supplementary Figure 9.** **The GSEA analysis for ferroptosis related RNA-seq data on AD-related gene sets**

(A) The enrichment plot of the GSEA analysis for ferroptosis related RNA-seq data (GSE126787) on AD-related gene sets (KEGG). (B) Heatmap of enriched terms across the core enrichment genes in GSEA analysis. Columns were colored by p-values, -log10(P)>10 were colored with gradient red, -log10(P)<10 were colored with gradient black.

**Supplementary Figure 10. The inhibitors of necroptosis and apoptosis could partially rescue the Aβ induced neuronal death**

(A) Primary neurons were treated with inhibitors of apoptosis (Emricasan, 10μM), necrosis (Nec-1, 50μM), ferroptosis (Lip-1,100 nM) and indicated vehicle for 24h. The cell viability was accessed by CCK8 assays (n=5). (B) Primary neurons were exposed to 20μm Aβ_1-42_ or combined with inhibitors of apoptosis (Emricasan, 10μM) / necrosis (Nec-1, 50μM) / ferroptosis (Lip-1,100 nM) for 24h. The cell viability was accessed by CCK8 assays (n=5). (C) The Fpn protein level in Aβ treated primary neurons. The quantitative data were listed below.

Data are shown as the mean ± SD of at least 3 independent experiments. Statistical analyses were carried out using mutiple t-tests. *p < 0.05; **p < 0.01; ***p < 0.001

**Supplementary Figure 11. Aβ injection induced iron accumulation in hippocampus**

(A) The tissue iron content (μg/g) in hippocampus exposed with Aβ. (B) The quantification for protein level of Fpn, FTH and Gpx4 in the hippocampus exposed with Aβ.

Data are shown as the mean ± SD of at least 3 independent experiments. Statistical analyses were carried out using mutiple t-tests. *p < 0.05; **p < 0.01; ***p < 0.001

**Supplementary Figure 12. Overexpression of Fpn in the hippocampus of *APP/PS1* mice showed no difference on basal locomotive behavior**

(A) Representative immuno-fluorescence staining of brain slice from the *APP/PS1* mice that were injected with AAV virus which overexpressed Fpn or control AAV virus in the hippocampus. Sections were stained for DAPI and the AAV were labeled with m-cherry. (B) The protein level of Fpn in the hippocampus of *APP/PS1* mice injected with AAV expressing Fpn or control virus. (C)The moved distance of *APP/PS1* mice injected with AAV in the Morris water maze (n= 10). (D) The percentage of time spent freezing during training or before the tone in the fear conditioning test of these mice (n=10).

Data are shown as the mean ± SD of at least 3 independent experiments. Statistical analyses were carried out using two-way ANOVA and mutiple t-test. *p < 0.05; **p < 0.01; ***p < 0.001

**Supplementary Figure 13. Original images of representative western blots in all the Figures**

**Supplementary Figure 14. Original images of representative western blots in all the Supplementary Figures**
