# Supplementary figures and images for: Loss of ferroportin induces memory impairment by promoting ferroptosis in Alzheimer’s disease
Source: Cell Death Differ. 2021 Jan 4;28(5):1548–62. doi: 10.1038/s41418-020-00685-9 (PMC8166828; doi:10.1038/s41418-020-00685-9)

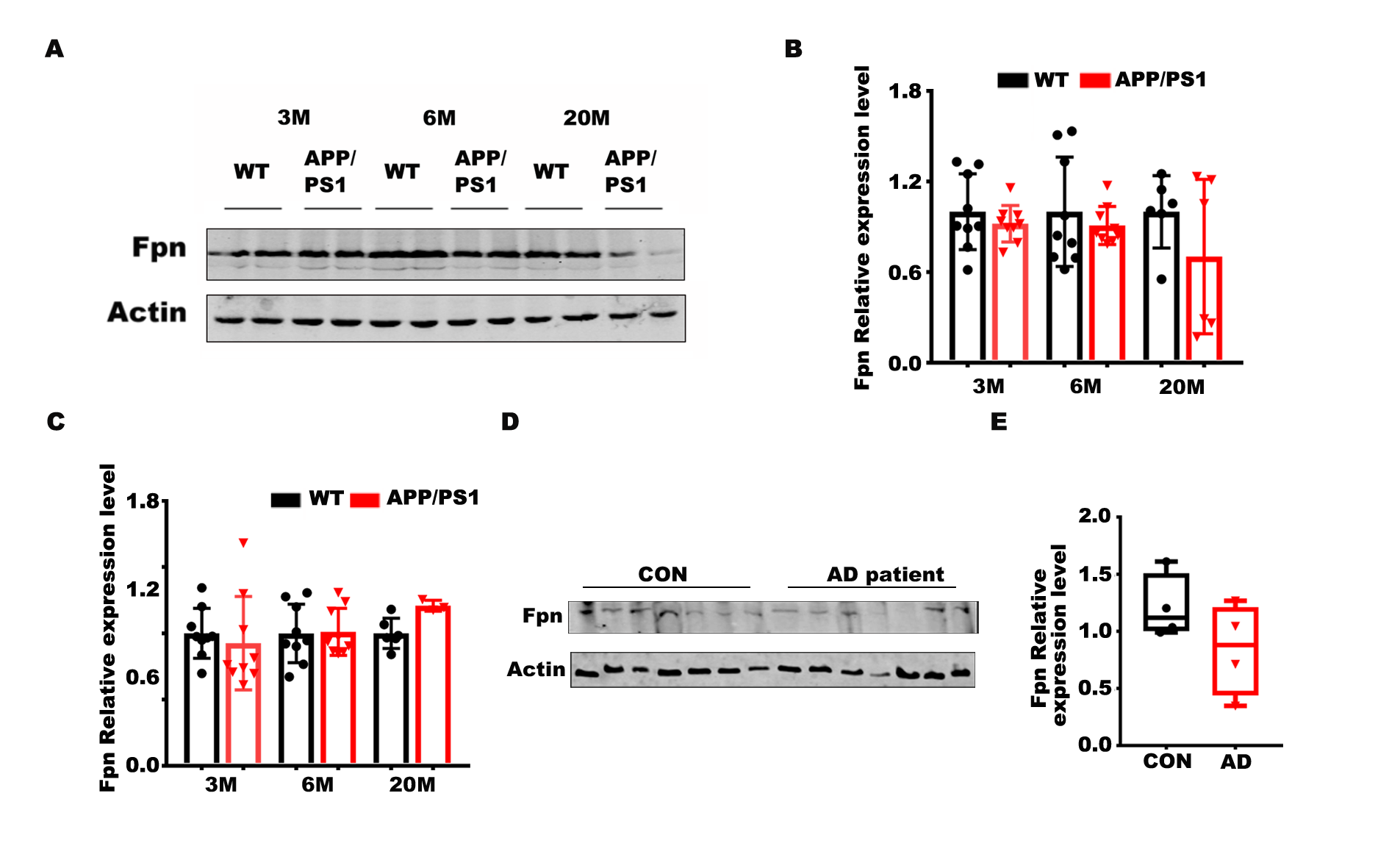

Supplement: Supplementary file 7 — Supplementary Fig. 1 [file 41418_2020_685_MOESM7_ESM.tif]

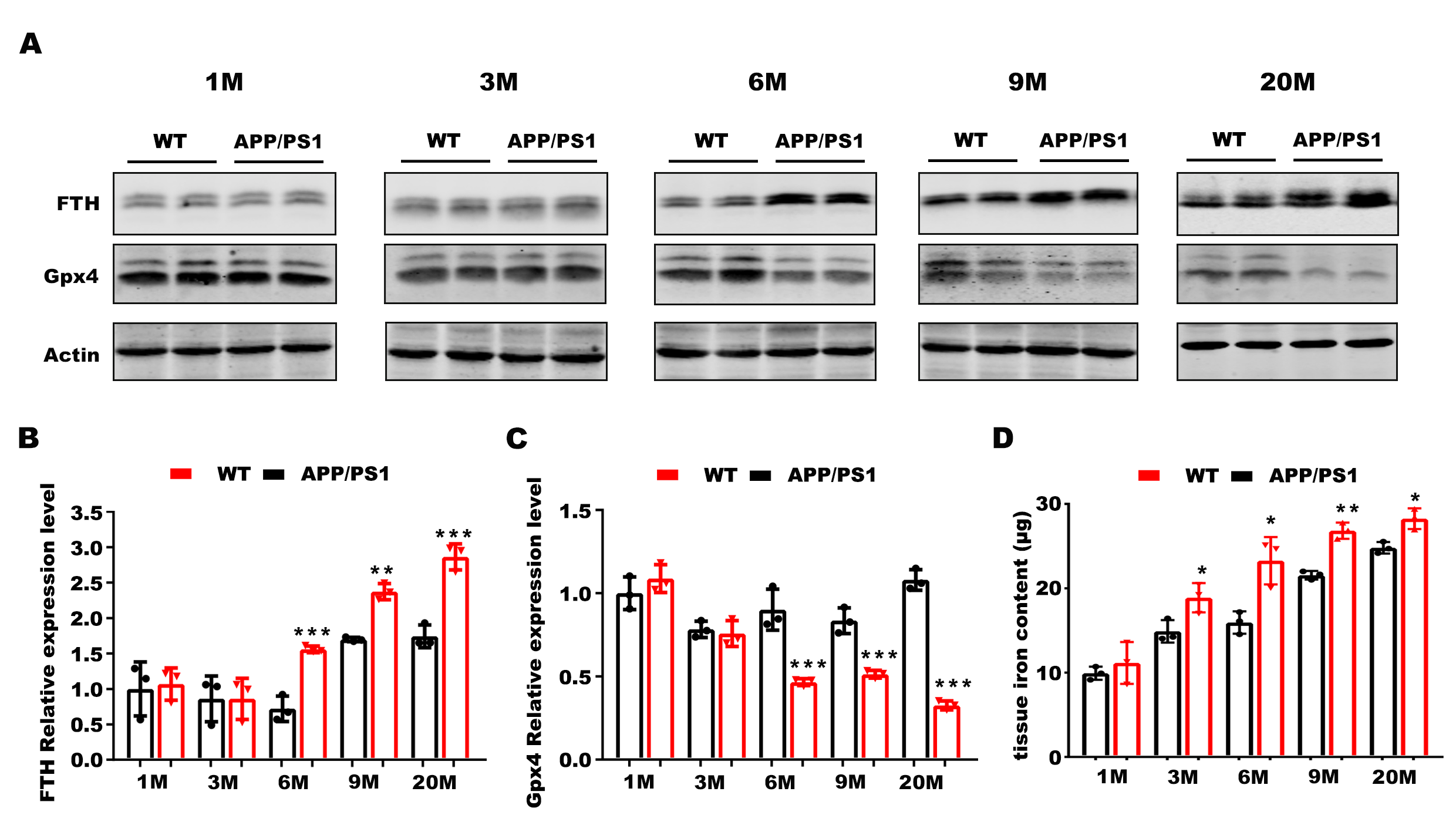

Supplement: Supplementary file 8 — Supplementary Fig. 2 [file 41418_2020_685_MOESM8_ESM.tif]

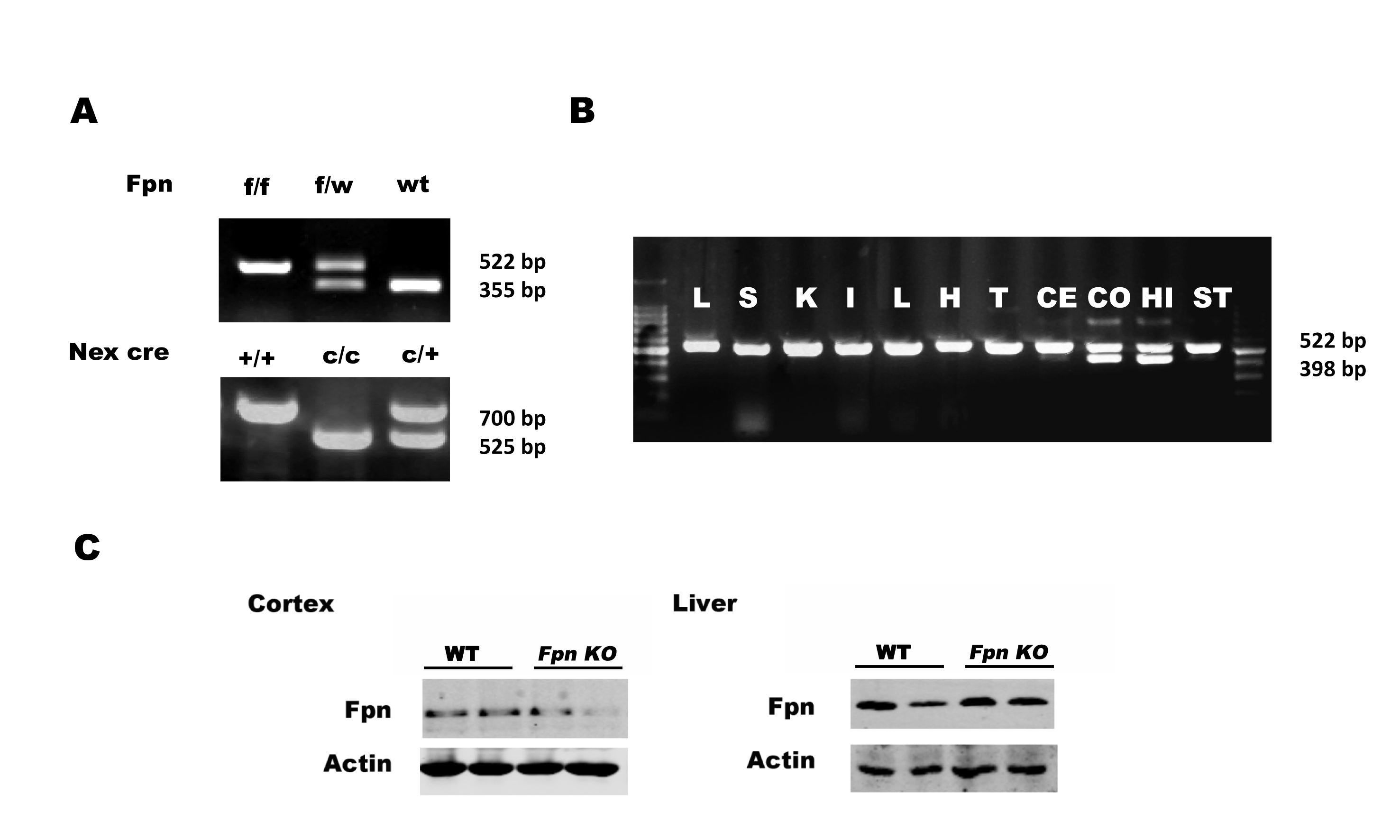

Supplement: Supplementary file 9 — Supplementary Fig. 3 [file 41418_2020_685_MOESM9_ESM.tif]

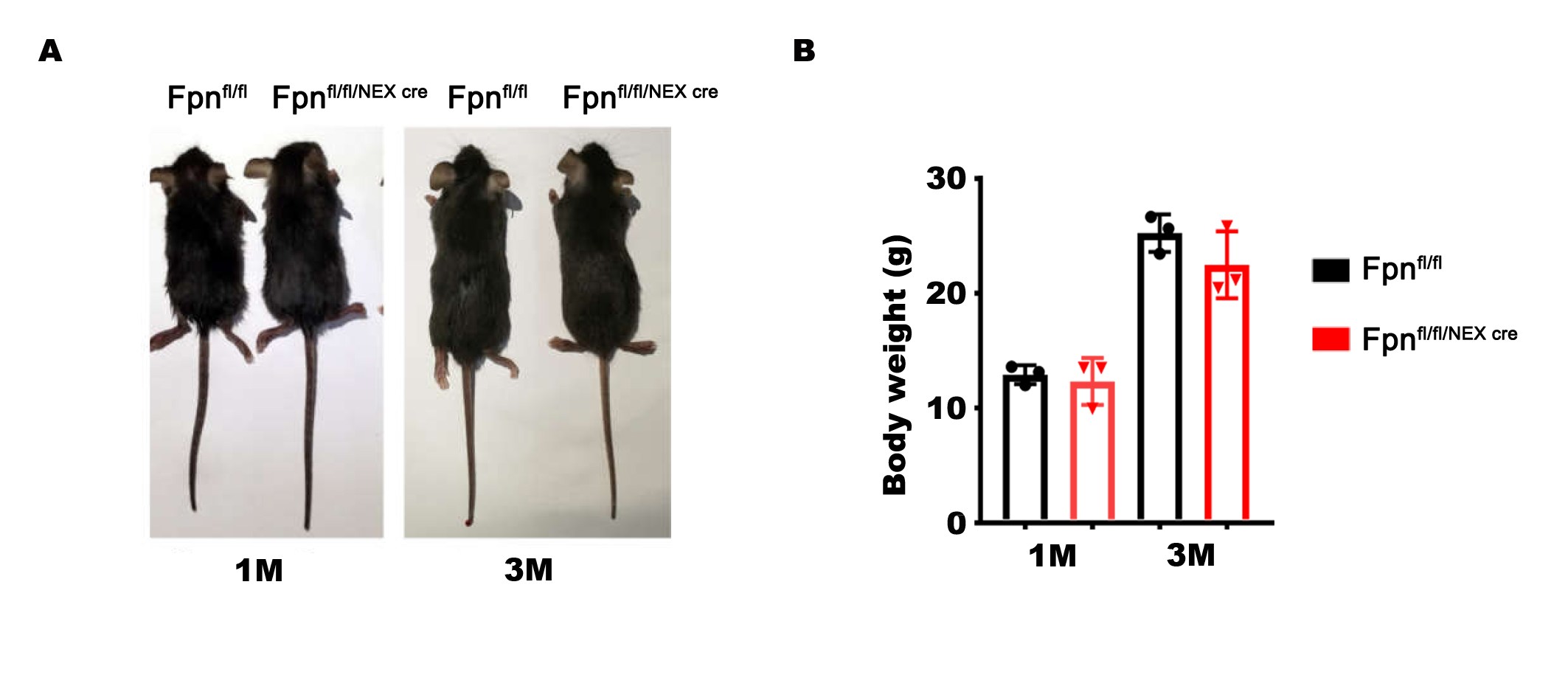

Supplement: Supplementary file 10 — Supplementary Fig. 4 [file 41418_2020_685_MOESM10_ESM.tif]

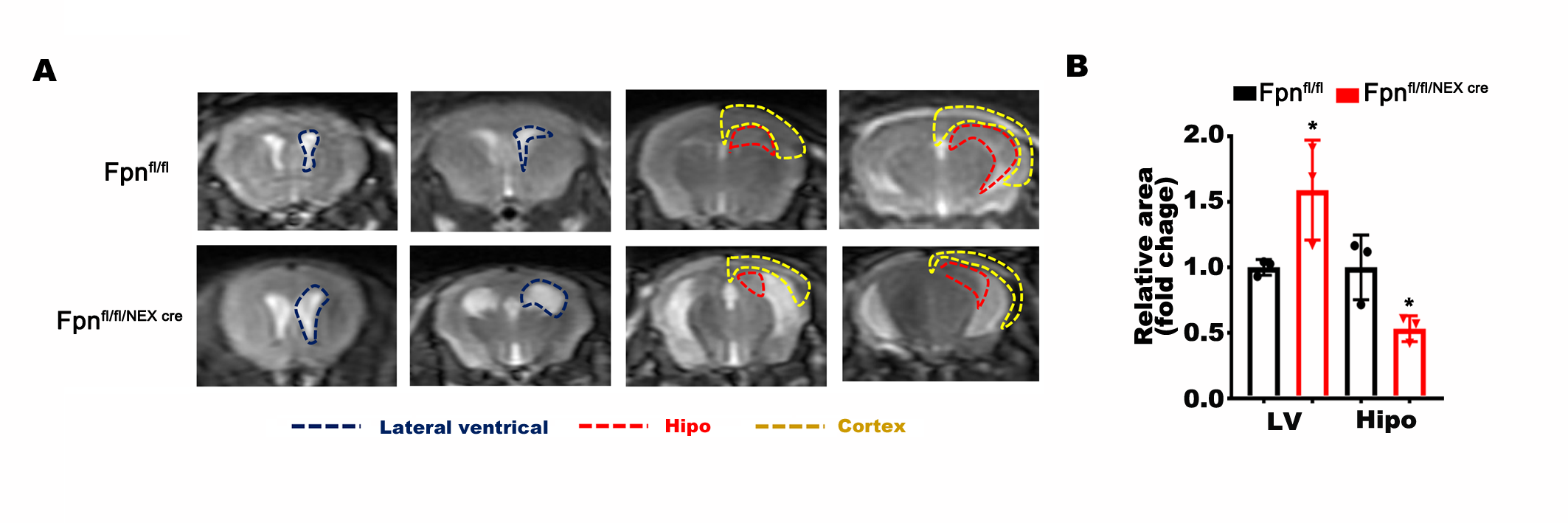

Supplement: Supplementary file 11 — Supplementary Fig. 5 [file 41418_2020_685_MOESM11_ESM.tif]

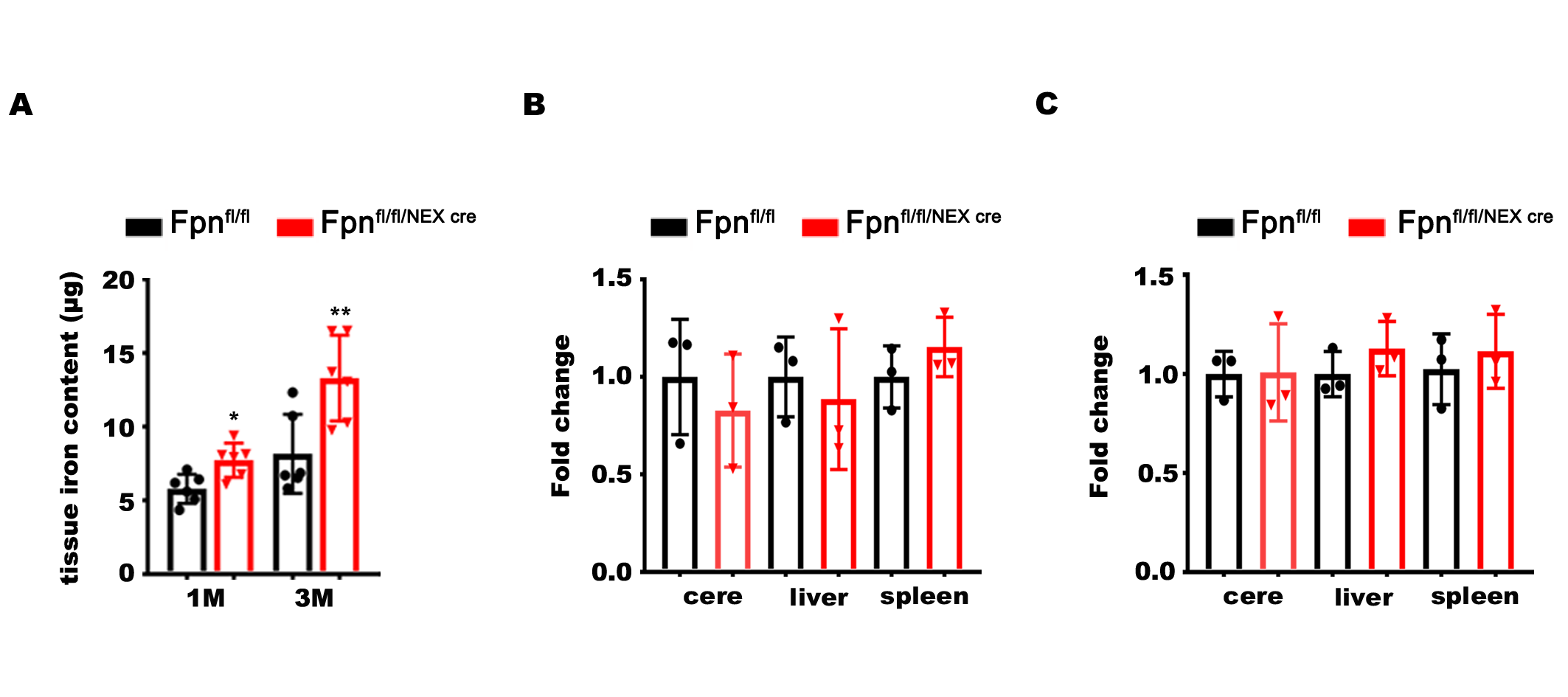

Supplement: Supplementary file 12 — Supplementary Fig. 6 [file 41418_2020_685_MOESM12_ESM.tif]

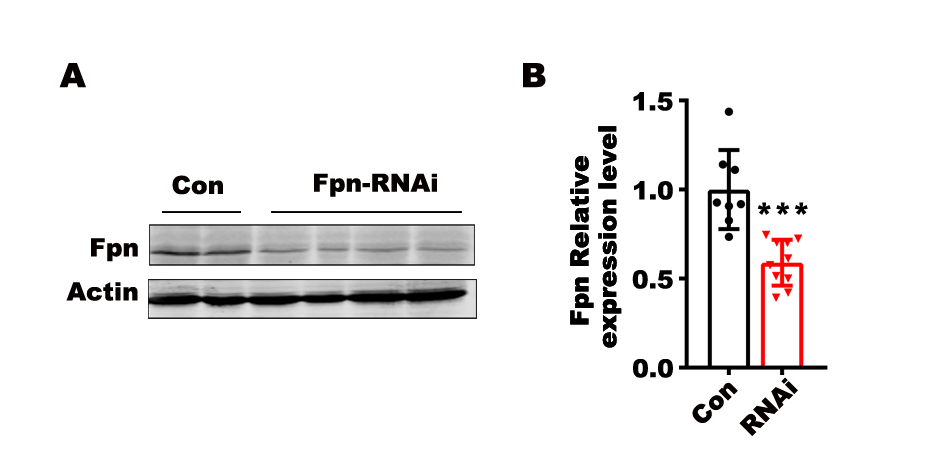

Supplement: Supplementary file 13 — Supplementary Fig. 7 [file 41418_2020_685_MOESM13_ESM.tif]

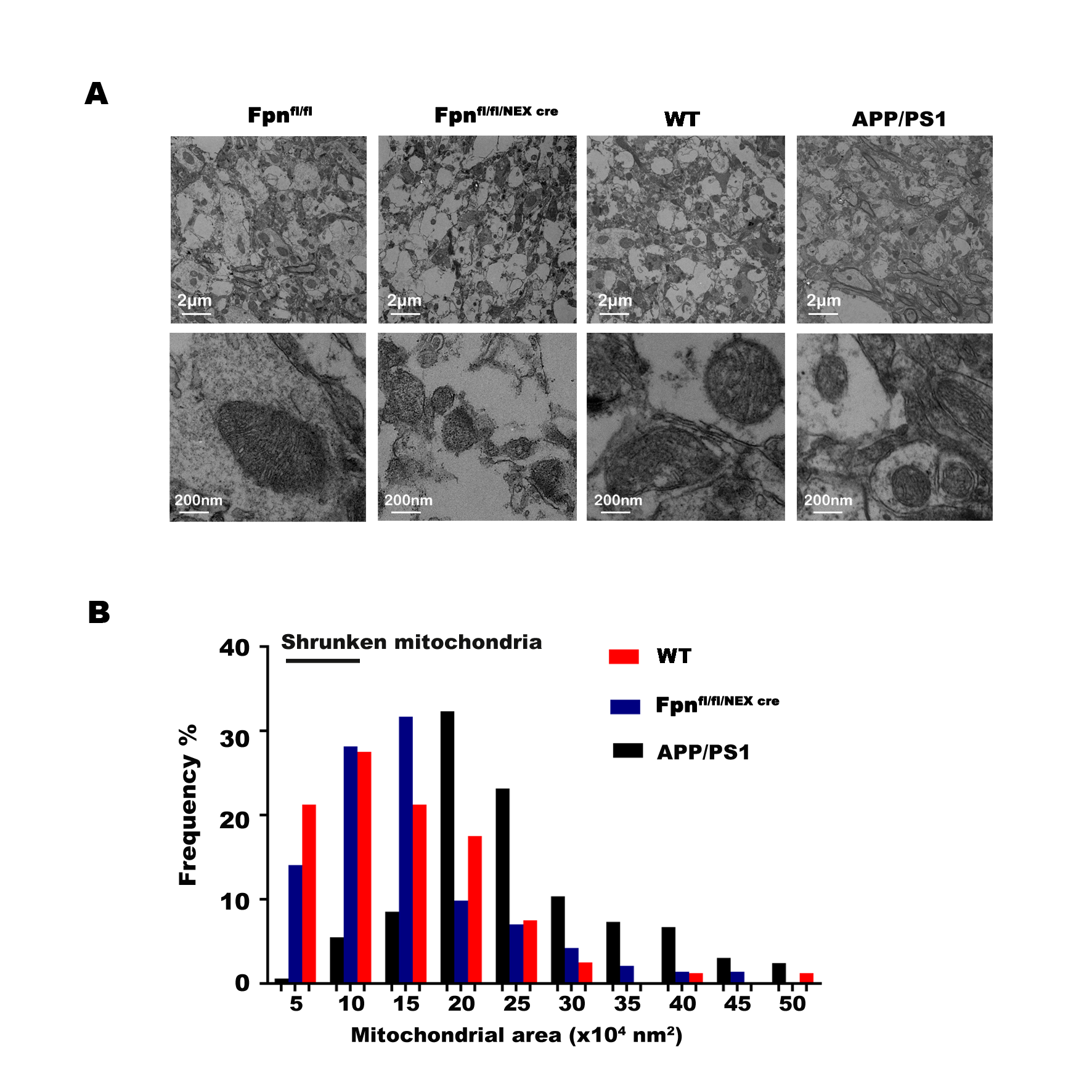

Supplement: Supplementary file 14 — Supplementary Fig. 8 [file 41418_2020_685_MOESM14_ESM.tif]

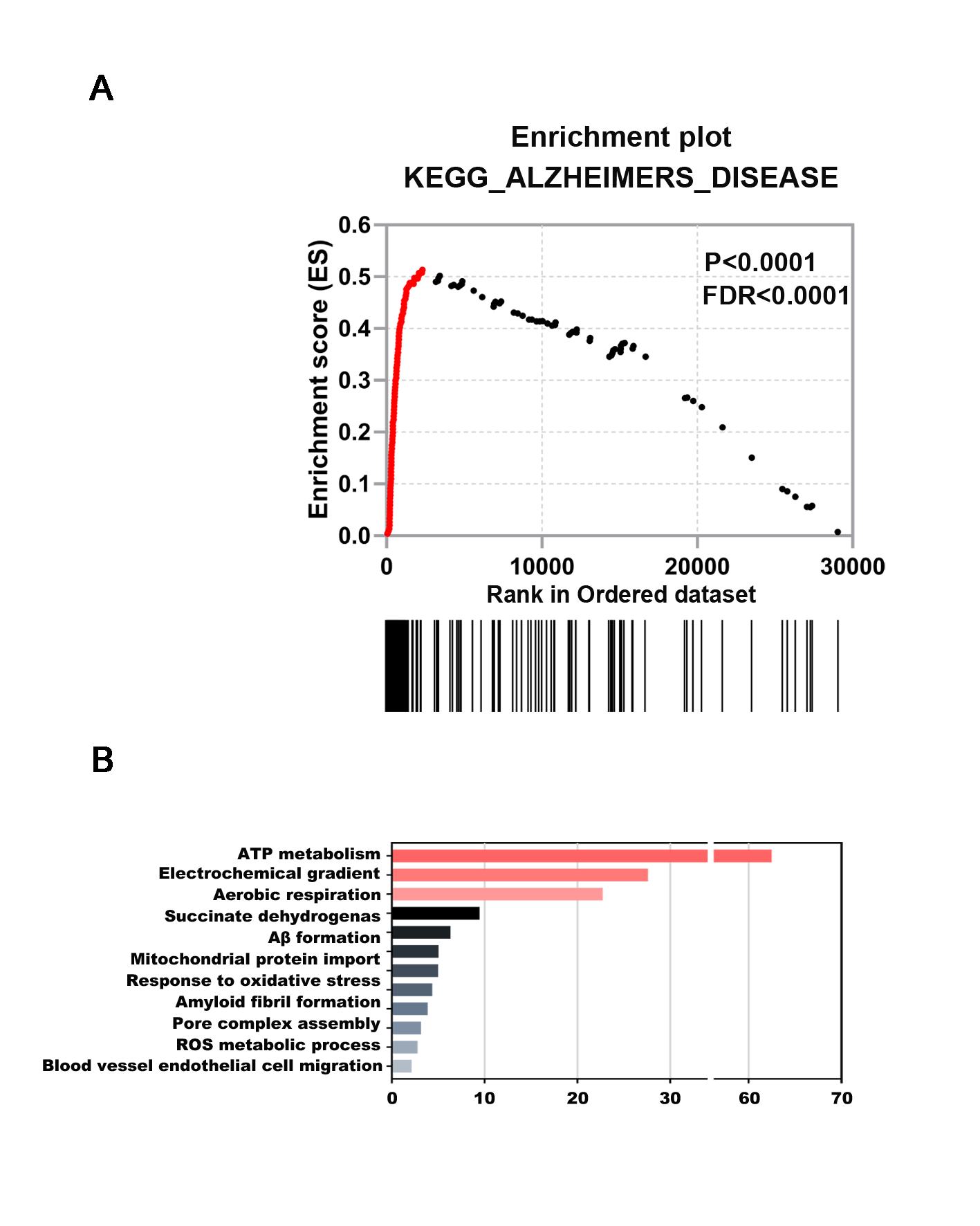

Supplement: Supplementary file 15 — Supplementary Fig. 9 [file 41418_2020_685_MOESM15_ESM.tif]

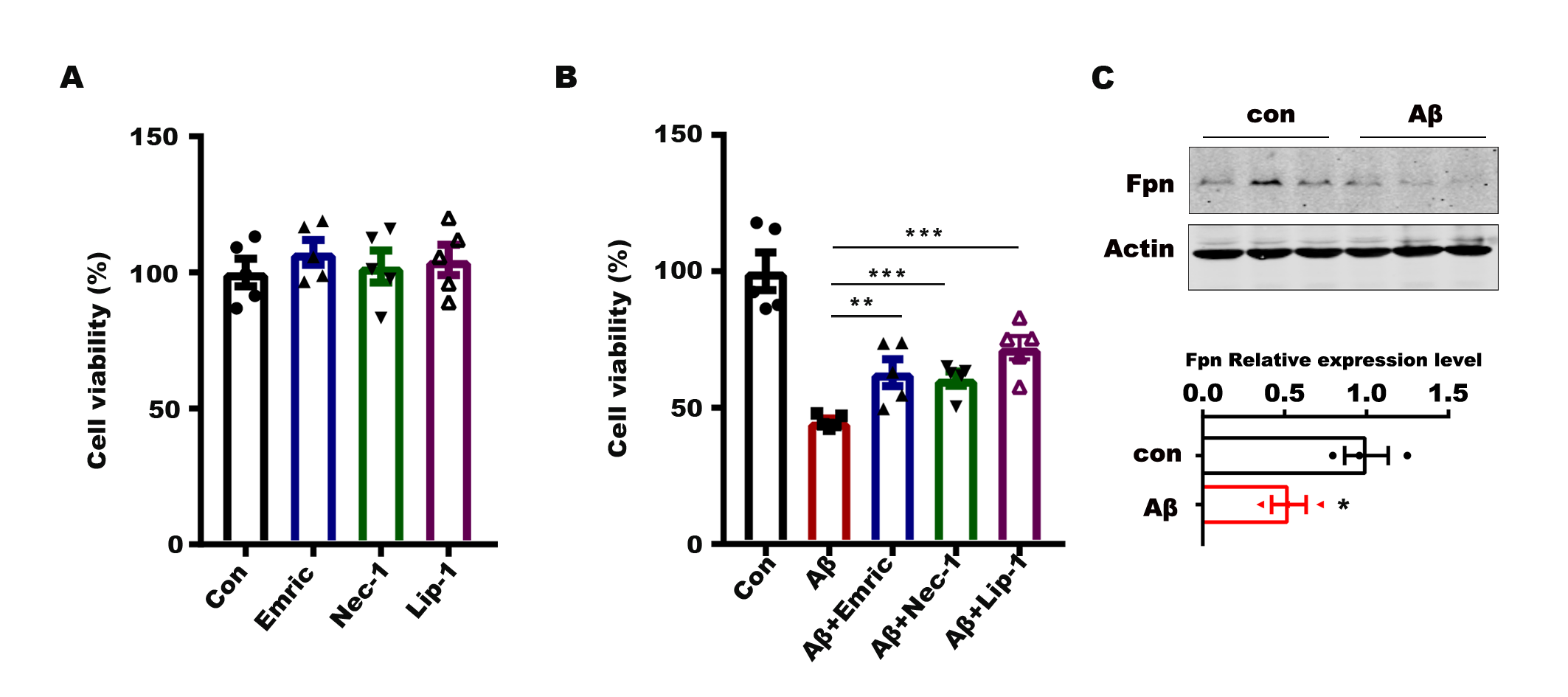

Supplement: Supplementary file 16 — Supplementary Fig. 10 [file 41418_2020_685_MOESM16_ESM.tif]

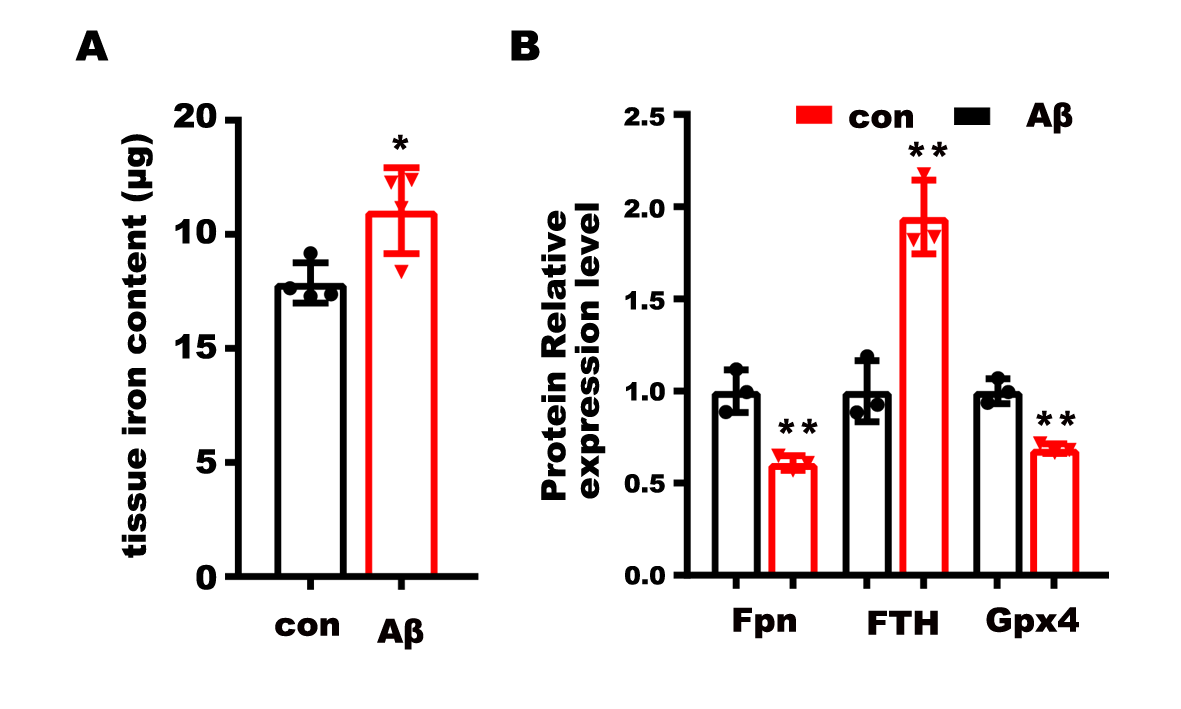

Supplement: Supplementary file 17 — Supplementary Fig. 11 [file 41418_2020_685_MOESM17_ESM.tif]

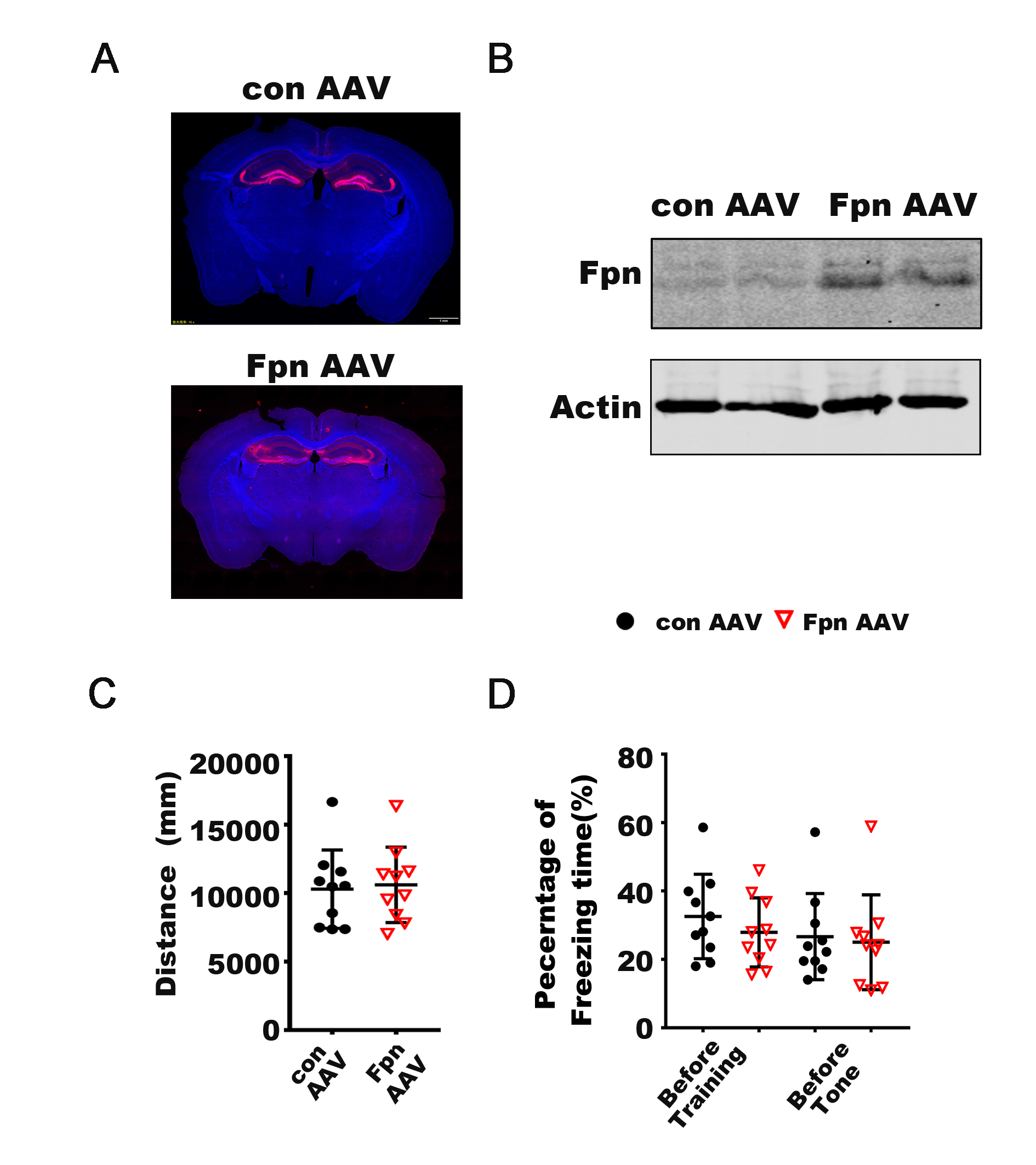

Supplement: Supplementary file 18 — Supplementary Fig. 12 [file 41418_2020_685_MOESM18_ESM.tif]

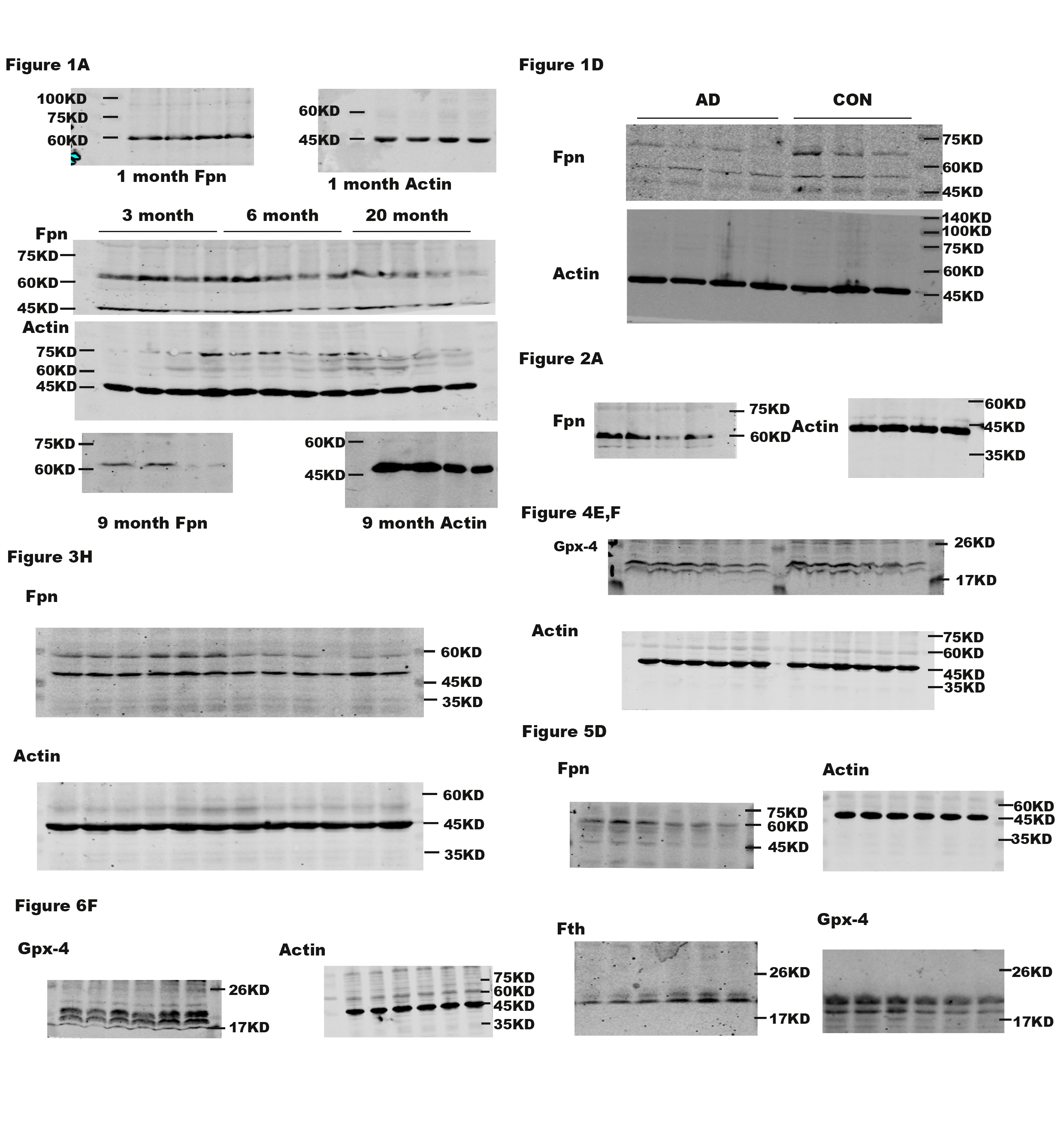

Supplement: Supplementary file 19 — Supplementary Fig. 13 [file 41418_2020_685_MOESM19_ESM.tif]

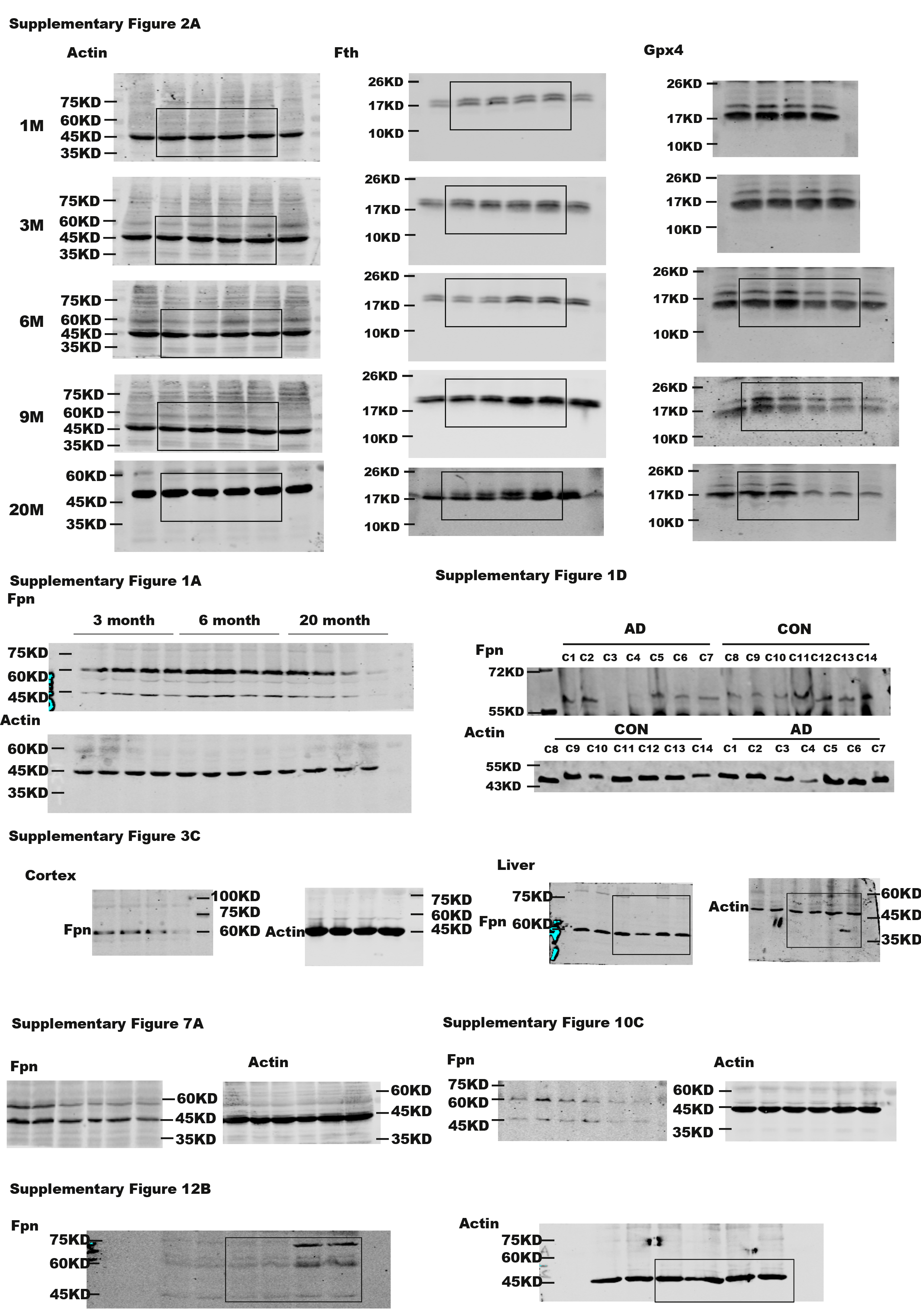

Supplement: Supplementary file 20 — Supplementary Fig. 14 [file 41418_2020_685_MOESM20_ESM.tif]
